# Well-Characterized Polyethyleneimine-/Carboxylated-Polyethylene-Glycol-Functionalized Gold Nanoparticles as Prospective Nanoscale Control Materials for In Vitro Cell Viability Assays: Particle Characterization and Toxicity Tests in Eight Mammalian Cell Lines

**DOI:** 10.3390/nano15020079

**Published:** 2025-01-07

**Authors:** Vytas Reipa, Vincent A. Hackley, Alessandro Tona, Min Beom Heo, Ye Ryeong Lee, Tae Geol Lee, Aaron Johnston-Peck, Tae Joon Cho

**Affiliations:** 1 Materials Measurement Laboratory, National Institute of Standards and Technology, Gaithersburg, MD 20899, USA; vincent.hackley@nist.gov (V.A.H.); alex.tona@nist.gov (A.T.); aaron.johnston-peck@nist.gov (A.J.-P.); 2 Division of Biomedical Metrology, Korea Research Institute of Standards and Science, Daejeon 34113, Republic of Korea; mbheo@kriss.re.kr (M.B.H.); yeryeong27@kriss.re.kr (Y.R.L.); tglee@kriss.re.kr (T.G.L.)

**Keywords:** gold nanoparticles, cell viability, assay controls, MTS, polyethylene imine, polyethylene glycol

## Abstract

The safety screening of manufactured nanomaterials (MNMs) is essential for their adoption by consumers and the marketplace. Lately, animal-based testing has been replaced by mechanistically informative in vitro assays due to the requirements of regulatory agencies. Cell viability assays are widely employed for manufactured nanomaterial hazard screening as a first-tier approach. Critical parts of such assays are positive and negative controls that serve as measurement benchmarks. We present the cellular viability and corresponding particle characterization obtained with eight different cell lines that were exposed to Au-PEI and Au-PEG-COOH nanoparticles. We showed that polyethyleneimine- and carboxylate-polyethylene-glycol-conjugated gold nanoparticles (AuPEI and Au-PEG-COOH) qualified for positive and negative controls in the in vitro cell viability assays used for MNM toxicological screening.

## 1. Introduction

Progress in nanotechnology has brought attention to the potential risks of novel materials to human health and the environment. The risks are related to the size-dependent physicochemical properties of manufactured nanomaterials (MNMs), resulting in altered transport across biological barriers and elevated reactivity due to the developed surface area [1]. Although nanotoxicology is a mature field, it is not realistic to expect full epidemiological and ecosystem data for every MNM product given the multiple specific hazard factors, such as the particle size, shape, crystallographic structure, and surface charge. Traditional toxicology is based on animal model safety screening, where hazard data are still lacking for 85% of major chemicals that have been in production for decades [2]. Over the last couple of decades, a drive by the regulatory agencies toward a non-animal-based testing recognized the increased role of mechanistically informative in vitro assays [3,4]. Tests developed for chemical hazard screening were adapted or modified for MNMs given their unique properties. Cell viability assays have been widely employed for MNM hazard screening as a first-tier approach. Several high throughput methods are used for determining the cell viability in vitro, such as 3-(4,5-dymethylthiazol-2-yl)-5-(3-carboxymehoxyphenyl)-2(4-sulphenyl)-2H-tetrazolium (MTS) [5], 3-(4,5-dimethylthiazo-2yl)-2,5-diphenyltetrazolium bromide (MTT) [6], (2,3-bis-(2-methoxy-4-nitro-5-suphonyl)-2Htetrazolium-5-carboxinilide) (XTT) [7], lactate dehydrogenase (LDH) [8], trypan blue exclusion [9], and neutral red assay [10]. The MTS protocol is based on the colorimetric change of tetrazolium salt upon its reduction by cytoplasmic reductase enzymes into formazan, which is detected by measuring the absorbance at 490 nm. Changes in the absorption intensity are directly proportional to the viable cell number, though assay conditions that affect reductase activity or possible MNM interferences must be taken into account. Gold nanoparticles can interfere with various assays due to their optical properties and chemical interactions with test reagents [11]; therefore, a careful analysis and/or control of such interferences should be included in the measurement procedure.

Essential parts of cell viability tests are positive and negative controls that serve as measurement references [12]. Controls are also included to demonstrate the correct assay performance and facilitate reproducibility, and are critical for data comparisons between testing laboratories [13,14,15]. It is customary to use toxicant unexposed cells in the growth media as a negative control, with subsequent normalization of the assay output to these background values. According to its definition, a negative control should elicit no cytotoxic response. A substance that provides known, significant, and consistent cellular damage for certain cell types in a particular assay can be utilized as a positive control, thus providing evidence that the assay is performing correctly. The control choice is also related to the assay endpoint and the exposure route.

At the early stage of nanotoxicology development, chemical controls were regularly used for in vitro MNM cytotoxicity assays, as testing protocols for established soluble chemicals were adopted [16]. In this way, many of the early studies related MNM-induced effects to those caused by chemicals, e.g., hydrogen peroxide or cadmium salts, such as CdSO_4_ for cell viability, oxidative stress, and genotoxicity [17,18]. For increased reliability and relevance, MNM hazards need to be assessed in at least three cell types by including chemical positive and negative controls [5]. Moreover, evidence has accumulated that MNMs behave differently with these assays due to their unique physicochemical properties, leading to unexpected biological effects, interferences with assay readouts, and reactivity with assay components [11,19]. In certain cases, MNM-based reference materials, such as amine-modified polystyrene nanoparticles (PSNPs) [20], have been reported as positive controls for cytotoxicity, oxidative stress, and genotoxicity [21,22]. Furthermore, positively charged PSNPs were even considered as positive controls for in vitro testing standardization; however, an interlaboratory study revealed that they lack sufficient colloidal stability [23]. Neutral or negatively charged PSNPs, as well as negatively charged gold nanoparticle (AuNP) materials (RMs 8011, 8012, and 8013; NIST, Gaithersburg, MD, USA), were also used as negative controls [24,25]. Therefore, up to now, there are no generally accepted nano-specific references that could be used as in vitro cytotoxicity assay controls, even though some selection criteria were defined [26,27]. Petersen et al. [26] listed three important characteristics of a positive control for in vitro assays—consistent performance, accessibility, and safety for the user and the environment. There are seven requirements listed to ensure a control’s consistent performance, in particular the biological mechanism of action, ease of preparation, chemical purity, verifiable physical properties, stability, ability to generate the full dynamic range for the assay, and known technical/biological interference.

A cationic charge was shown to result in an increased nanoparticle cytotoxicity [28]. Nevertheless, as demonstrated for PSNPs, in the presence of serum proteins, NPs are covered with a protein corona and their zeta potential shifts toward a neutral value, regardless of their native charge measured in water [29]. AuNPs have been the most widely employed MNMs for biomedical applications due to their low cytotoxicity [30], high chemical and colloidal stability, and flexibility in chemical modification [31,32]. It was established that the particle surface charge, hydrophobicity, and size are the main factors governing the toxicity of AuNPs [16]. In particular, the conjugation of polycations to AuNPs gives them a positive charge that ensures an electrostatic interaction with the negatively charged cellular membranes and eases the particle cellular internalization [33]. Polyethyleneimine (PEI), also a polycation, is highly water soluble and is routinely used in gene transfection/delivery applications [34,35]. Previously, we identified material properties critical to PEI-conjugated NP (Au-PEI) stability in biological media [36,37,38] and reported a notable dose-proportional toxicity to several mammalian cells and frog embryos, which suggests their potential utility as a positive control for in vitro and in vivo nanotoxicity assays [38].

In this report, we present data on the cellular viability and corresponding particle characterization obtained with eight different cell lines that were exposed to Au-PEI and carboxylate-polyethylene-glycol-conjugated AuNPs (Au-PEG-COOH, as anionic NPs). Then, we discuss their qualification for positive and negative controls when applied in the in vitro cytotoxicity screening of MNMs in the context of previously defined requirements for such applications [13,25].

## 2. Materials and Methods

Certain commercial equipment, instruments, or materials are identified in this report to adequately specify the experimental procedure. Such identification does not imply recommendation or endorsement by the National Institute of Standards and Technology, nor does it imply that the materials or equipment identified are necessarily the best available for the purpose.

### 2.1. Materials

Cadmium sulfate (CdSO_4_), gold III chloride hydrate (HAuCl_4_∙3H_2_O), and branched 25 kDa PEI (bPEI25kDa) were purchased from Sigma Aldrich (St. Lois, MO, USA). SH-PEG5k-COOH was obtained from Biopharma PEG (Watertown, MA, USA). Cell growth media, including phosphate buffered saline (PBS), Dulbecco’s Modified Eagle’s Medium (DMEM), Eagle’s Minimum Essential Medium (EMEM), and F12 were obtained from the ATCC (Manassas, VA, USA). Optimized DMEM was provided by AddexBio (San Diego, CA, USA). Iscove’s Modified Dulbecco’s Medium (IMDM) and Roswell Park Memorial Institute (RPMI) medium were sourced from Life Technologies (Grand Island, NY, USA).

### 2.2. Nanoparticle Synthesis

Au-PEI nanoparticles (Au-PEI@NIST) were synthesized by reducing aqueous HAuCl_4_ solution with 25 kDa aqueous branched PEI, as described previously [38,39]. Briefly, 10 mL of aqueous bPEI25 kDa (10% mass fraction) was added to 100 mL of aqueous HAuCl_4_ (2.5 mmol/L) at room temperature (r.t.) and heated up to 80 °C using an oil bath with stirring. After reaching 80 °C, the reaction mixture was then stirred for an additional 3 h. The oil bath was then removed to allow the reaction mixture to cool down to room temperature (22 °C to 24 °C) and purified using stirred-cell ultrafiltration with an Amicon 100 kDa MWCO regenerated cellulose membrane (MilliporeSigma, Bedford, MA, USA). In this step, the reaction medium was reduced from 100 mL to 10 to 20 mL, then backfilled with deionized water (DIW) to about 100 mL. This purification cycle was repeated 4 times, with the final volume of Au-PEI@NIST suspension adjusted to ≈100 mL with DIW, which yielded an Au concentration of ≈2.5 mmol/L (or 500 µg/mL).

PEG-COOH stabilized AuNPs (Au-PEG-COOH@NIST) were prepared by a modified method of a previous study [38] as follows: 100 mL of aqueous HAuCl_4_ (0.25 mmol/L) was heated to a reflux. To this reflux, 1 mL of aqueous sodium citrate (120 mmol/L) was added and stirred for 30 min. The red suspension was cooled down to room temperature, then 1 mL of aqueous SH-PEG-COOH (5 mmol/L, molar mass 5 kDa) was added and stirred for an additional 3 h at r.t. The reaction mixture was purified by Amicon ultra15, RC membrane, MWCO 100 kDa centrifugal filtration (MilliporeSigma, Bedford, MA, USA). The final product concentration was adjusted to be similar to the Au-PEI@NIST by reducing the final volume to 10 mL (≈2.5 mmol/L or 500 µg/mL of Au). Besides the lab-made Au-PEI@NIST and Au-PEG-COOH@NIST, commercially available Au-PEI@Cs from Nanopartz Inc. (Loveland, CO, USA) and nanoComposix (San Diego, CA, USA) and Au-PEG-COOH@C (Nanopartz Inc.) were also obtained for comparison purposes in terms of the physicochemical properties, colloidal stability, and cytotoxicity. These commercial sources are referred to as C1 and C2 in no particular order.

### 2.3. Particle Characterization

Measurement methods and instrumentation for physicochemical property characterizations, such as UV–Vis absorbance, dynamic light scattering (DLS), zeta potential (ZP), transmission electron microscopy (TEM), and stability tests are described in the Supporting Information (SI). For the DLS, ZP, and TEM measurements; stability test; and in vitro study, the final concentration of the AuNPs@NISTs in suspension, referred to here as the ‘*test suspension*’, should be around 50 µg/mL and diluted by appropriate media, such as DIW or physiological media.

### 2.4. Cell Lines, Growth Conditions, and Treatments

Before the treatment with the Au NPs and controls, the cells were seeded into 96-well plates at a density of 1 × 10^4^/well.

Chinese Hamster Ovary (CHO K1) cells were purchased from the ATCC (Manassas, VA, USA) and cultured under standard conditions (grown for 24 h at 37 °C, 95% humidity) in IMDM, supplemented with 10% fetal bovine serum (FBS; 26140-079, GIBCO, NY, USA) and 1% penicillin/streptomycin PennStrep, (ThermoFisher, Waltham, MA, USA).

Lung carcinoma (A549) cells were obtained from the ATCC and cultured in RPMI medium with 10% FBS and 1% PennStrep (ThermoFisher, Waltham, MA, USA).

Epithelial Adenocarcinoma (HeLa) cells were purchased from the ATCC, and cultured in EMEM, supplemented with FBS and 1% PennStrep (ThermoFisher, Waltham, MA, USA).

Mouse embryo fibroblasts (NIH 3t3s) were obtained from the ATCC and grown in DMEM, modified with 4 mM L-glutamine, containing 10% FBS and 1% PennStrep.

Human keratinocytes (HaCaTs) were sourced from AddexBio (San Diego, CA, USA), grown in AddexBio Optimized DMEM + 10% FBS and 1% PennStrep (ThermoFisher, Waltham, MA, USA).

Human malignant melanoma (SK-MEL-28) cells were provided by the ATCC and grown in EMEM, supplemented with 10% FBS and 1% PennStrep (ThermoFisher, Waltham, MA, USA).

Human Bronchial Epithelial cell line (BEAS-2B) and Human liver cancer cell line (HepG2) cells were purchased from the ATCC. They were cultured in DMEM (11995073, GIBCO, NY) containing 10% FBS and 1% PennStrep (ThermoFisher, Waltham, MA, USA).

Following the cell growth in 96-well plates (Falcon Tissue Culture Plate, Flat bottom from Corning, Durham, NC, USA) for 24 h, the growth media was removed, and a series dilution of AuNPs@NIST was loaded in triplicate, together with a chemical control CdSO_4_ in the growth medium, as appropriate for each specific cell type. The cells were treated with seven serial dilution concentrations of the Au-PEI@NIST (0 μg/mL–12 μg/mL), Au-PEG-COOH@NIST (0 μg/mL–50 μg/mL), and CdSO_4_ (0–20 μg/mL). Before incubation with cells, the test substance working solutions were prepared in the cell-specific medium without FBS by vortexing and a brief bath sonication for better dispersion. The AuNP concentrations were measured using the absorbance at the plasmon peak (518 nm < λ < 523 nm, Table 1) and corresponding extinction coefficients [40].

### 2.5. Testing of PEI Release from Au-PEIs

The presence of trace amounts of PEI in the test solution may distort the toxicity assessment of AuPEI@NIST, resulting in a false positive reading. There could be residual PEI left over from the particle synthesis procedure, or it can be released from the Au-PEI@NIST shell. To check for a such a possibility, we tested the robustness of the gold NP PEI shell by conducting a centrifugal filtration (CF) of the Au-PEI@NIST, and then examined for free PEI. The toxicity of the post-centrifugation Au-PEI@NIST was evaluated and compared with the original particles using the CHO K1 cells. An identical aliquot (10 mL, ≈50 µg/mL) of Au-PEI@NIST was centrifugally filtered (CFed) using Amicon Ultra 15 (regenerated cellulose membrane, molar mass cut off 50 kDa, MilliporeSigma, Bedford, MA) with a Beckman J2 centrifuge (3020× *g* or 5000 rpm, Beckman Coulter, Indianapolis, IN) for 20 min per each centrifugation. After the first centrifugation cycle, the residual AuNP inside the filter (less than 200 µL) was re-dispersed back into 10 mL of DIW. Then, 2 mL of the 1st re-dispersed AuNP was transferred for the toxicity evaluation and the remaining 8 mL AuNP dispersion was repeated by an identical centrifugation process 4 more times. Eventually, a 5th CF cycle was performed to obtain the last 2 mL of 5th CFed Au-PEI@NIST sample (total of 5 CFed samples, 2 mL each). The filtrate toxicity was tested with CHO K1 cells (Figure S12).

### 2.6. MTS Assay

The cytotoxicities of the Au-PEI@NIST and Au-PEG-COOH@NIST for the CHO, HaCaT, A549, 3T3, HeLa, SK-Mel-28, BEAS-2B, and Hep G2 cells were tested with the MTS (3-(4,5-dimethylthiazol-2-yl)-5-(3-carboxymethoxyphenyl)-2-(4_sulphophenyl)-2H-tetrazolium) assay CellTiter 96 Aqueous, obtained from Promega (Madison, WI, USA). A stock solution of MTS was diluted 5x in the appropriate colorless medium without a serum and antibiotic. The optical density of the soluble formazan, which is a product of reductase-reduced tetrazolium dye, was correlated with the cell count. The measurement procedure was adopted from [41]. Following the 24 h exposure of cells to AuNPs and controls, the supernatant was decanted, and the wells were rinsed twice with warm PBS. A total of 100 μL of MTS solution was pipetted into each well, followed by a 1 h to 2 h incubation at 37 °C, 95% humidity, and 5% CO_2_. The absorbance was recorded at 490 nm using a Synergy 5 plate reader (BioTek, Winoski, VT, USA). The cell viability was calculated using viability (%) = [(Experimental A_490nm_ − Residual _A490AuPEI@NIST_ − Background A_490nm_)/(Negative control A_490nm_ − Background A_490nm_)] × 100.

The experimental A_490_ reading was adjusted for the residual _A490AuPEI@NIST_ absorbance following two rinses with warm PBS. The background A_490_ absorbance reading was recorded in empty wells, while the negative control A_490_ was recorded in the wells that contained transparent cell growth media.

The error bars on the dose–response graphs indicate one standard deviation from three technical replicate measurements.

**Table 1:** Physical properties of AuNPs@NIST and commercial AuNPs.

*AuNPs*	*D*_z_ (nm) *^a^*	*D*_TEM_ (nm) *^b^*	*ZP* (mV) *^a^*/pH	*SPR* (nm) *^a^*	*Mass Ratio* (PEI/Au)
Au-PEI@NIST	25.7 ± 0.4	11.5 ± 1.8	+15.6 ± 0.9/9.5	521	4.9~5.6 *^c^*
Au-PEG-COOH@NIST	35.4 ± 0.3	14.3 ± 0.9	−18.8 ± 4.0/6.8	521	NA
Au-PEI@C1	22.4 ± 0.3	10.0 ± 1.0	−10.9 ± 0.5/10.9	519	NA
Au-PEI@C2	31.4 ± 0.7	10.5 ± 1.0	+35.1 ± 0.5/7.4	523	NA
Au-PEG-COOH@C1	38.8 ± 0.9	10.0 ± 0.8	−40.5 ± 2.0/6.4	518	NA

*a:* in DI water. *b*: TEM data of Au-PEI@NIST and Au-PEG-COOH@NIST were obtained from 739 and 982 particles, respectively. Commercial AuNPs properties were provided by manufacturers. *c*: [38,42].

### 2.7. Testing the Optical Interference of AuNPs with the MTS Assay

Several potential modes of NP interference with the MTS assay were proposed [19]. A small amount of Au-PEI@NIST NPs may penetrate cells and adhere to the cell membrane, as well as to the plate well bottom following the decanting of the NP suspension and subsequent rinses with warm PBS (see above). As AuNPs absorb at 490 nm (the readout of MTS assay), this may elevate the final absorbance readout and misrepresent the assay result. To account for this, we measured the plate absorbance at 490 nm following the incubation of the Au-PEI@NIST with the CHO K1 cells for 24 h and after consecutive warm PBS rinses up to five times.

### 2.8. Statistical Analysis

ANOVA was performed with Sigmaplot 12.0 (SPSS, Chicago, IL, USA) for the MTS cytotoxicity assays to determine statistical differences using *p* < 0.05. The IC_50_ values were calculated from the dose–response data by fitting them to a sigmoidal function using Sigmaplot 12.0.

## 3. Results

### 3.1. Physicochemical Properties

The physicochemical properties of the in-house-synthesized AuNPs@NIST (Au-PEI@NIST and Au-PEG-COOH@NIST) were characterized by multiple measurement techniques, including DLS for the z-average hydrodynamic diameter (*D*_z_), electrophoretic light scattering for the zeta potential (ZP), TEM for the core diameter (*D*_TEM_), ultraviolet-visible spectroscopy (UV–Vis) for the surface plasmon resonance (SPR) band position, and thermogravimetric analysis (TGA) for the surface coverage of the Au-PEI@NIST (mass ratio of PEI to Au). A detailed surface chemistry study of the Au-PEI@NIST was conducted by attenuated total reflectance Fourier transform infrared (ATR-FTIR) spectroscopy and X-ray photoelectron spectroscopy (XPS) and described in a previous report [36,37]. Representative measurement data for the AuNPs@NIST and commercially sourced AuNPs (Au-PEI@C1, Au-PEI@C2, and Au-PEG-COOH@C1) are presented in Table 1. Additional data regarding the particle sizes, ZP values, SPRs, and pHs of the AuNP suspensions in biological media were also assessed and are listed in Table S2.

**Figure 1:**
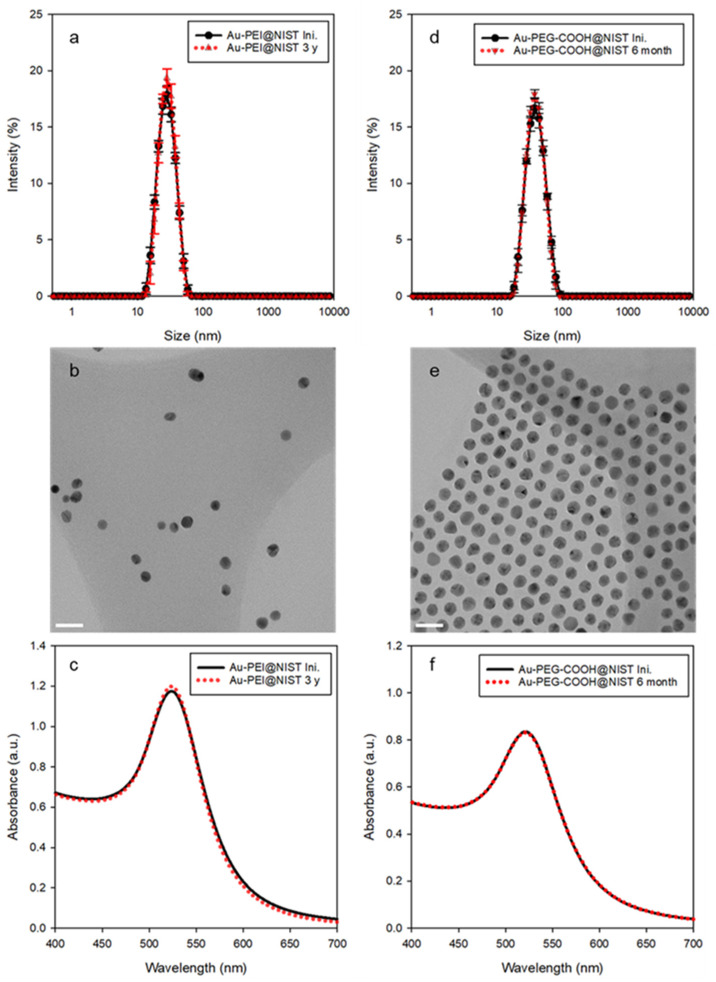
Comparison of representative property data for the two AuNPs@NIST formulations: (**a**,**d**) intensity-weighted size distributions by DLS, (**b**,**e**) TEM images (scale bars are 30 nm), and (**c**,**f**) SPR bands by UV–Vis for the Au-PEI@NIST and Au-PEG-COOH@NIST, respectively. Red dots indicate data obtained for particles aged in DIW for up to three years.

DLS and TEM were used to assess the hydrodynamic particle size in solution and Au core size as deposited on a substrate, respectively. As shown in Table 1, the z-average diameter (*D*_z_) values of the Au-PEI@NIST and Au-PEG-COOH@NIST, obtained by DLS measurements, were 25.7 ± 0.4 nm (Figure 1a) and 35.4 ± 0.3 nm (Figure 1d), respectively. On the other hand, the TEM revealed the core sizes (*D*_TEM_) of the Au-PEI@NIST and Au-PEG-COOH@NIST as 11.5 ± 1.8 nm (Figure 1b) and 14.3 ± 0.9 nm (Figure 1e), respectively. The DLS size was larger because it provided an equivalent-sphere hydrodynamic diameter of the particle that included both the core and the organic shell, while the TEM imaged only the Au core. The ZP reflects the surface charge properties of the suspended particles in a specific medium. The ZPs of the Au-PEI@NIST and Au-PEG-COOH@NIST were +15.6 ± 0.9 mV and −18.8 ± 4.0 mV at pH ≈ 9.5 and pH ≈ 6.8, respectively, indicating cationic and anionic charges due to the PEI and PEG-COOH coatings. The UV–Vis absorbance spectrum for AuNPs typically showed an SPR peak near λmax ≈ 520 nm, and this value could vary from slightly below 520 nm to more than 535 nm, depending on the size of the AuNPs. The maximum absorption (*λ*_max_) of the SPR band for both the Au-PEI@NIST and Au-PEG-COOH@NIST was centered at 521 nm (Figure 1c,f), which is typical for spherical AuNPs in this size range [40].

The surface coverage of the AuPEI@NIST (mass ratio of PEI to Au) was determined by TGA (described in [38,42]). The mass ratio of PEI to Au in the Au-PEI conjugate was equal to 5.60 ± 0.24 (one standard deviation), which corresponded to a PEI/Au molar ratio of 24.7 (calculated using 42 as the unit molar mass of PEI) in the Au-PEI@NIST. This value indicates that ≈28% of the introduced PEI mass was conjugated after the reduction of Au^III^.

### 3.2. Colloidal Stability

Regarding physical/structural stability for the shelf-life (aging process described in the Supplementary Materials), the colloidal stability in different media, as well as over a broad pH range and temperature range, is vital for understanding the potential biomedical applications of AuNPs. These factors were assessed under various conditions utilizing an established protocol [43]. The Au-PEI@NIST yielded almost identical size distribution profiles and SPR band shapes preceding and following aging (Figure 1a,c; black line for initial and red dotted line for the sample aged 3 years), with no detectable morphological change induced by, for example, agglomeration or aggregation. This confirmed the outstanding long-term stability when stored at room temperature without the aid of any additional agents. Likewise, based on the UV–Vis measurements over time, the Au-PEG-COOH@NIST showed an adequate shelf-life up to 6 months (Figure 1d,f). In general, the observance of a red shift in the SPR peak suggests the formation of particle agglomerates or aggregates. However, there was no such observation for both the Au-PEI@NIST and Au-PEG-COOH@NIST, and its absence suggests resistance to agglomeration during the stability test.

The colloidal stability of the Au-PEI@NIST in physiologically relevant conditions was studied previously [42,43]. A freshly conducted stability test for this study is described in Figure S4.

The colloidal stability of commercial AuNPs were evaluated for comparison (detailed in Supplementary Materials, Figure S6). Two Au-PEIs with identical core sizes by TEM from different manufacturers, C1 and C2, were selected and tested. Only one type of Au-PEG-COOH was obtained from C1 for this study.

In summary, based on the UV–Vis absorbance, the Au-PEI@NIST was stable in PBS, showed moderate agglomeration without core fusion in DMEM, and had improved stability when FBS was included in DMEM. On the other hand, the Au-PEG-COOH@NIST was stable in PBS, showed a substantial loss of Au mass in DMEM (agglomeration was not clearly evident in DMEM) and some improvement (reduced loss/agglomeration) when FBS was present in DMEM. The Au-PEI@NIST showed exceptional colloidal stability over the entire pH range (in 50 mmol/L HCl (pH 1.2) and 50 mmol/L NaOH (pH 12)) for at least 24 h (Figure S5a–c), while the Au-PEG-COOH@NIST (Figure S5d–f) was destabilized in extremely acidic or basic solutions.

As shown in Figure S6, all the commercial AuNPs were stable for 48 h in PBS (Figure S6a–c), regardless of the surface ligand type. However, in DMEM, all the AuNPs experienced reduced colloidal stability (Figure S6d–f) in contrast to PBS, where the stability was comparable with the AuNPs@NIST. The Au-PEG-COOH@C1 was somewhat more stable in DMEM compared with the Au-PEI@C1 and Au-PEI@C2, but its SPR absorbance also decreased, as was observed with the Au-PEG-COOH@NIST. The stability tests of the commercially sourced particles in FBS-DMEM (Figure S6g–i) showed similar trends to the AuNPs@NIST. Also, the commercially sourced Au-PEIs had diminished stability in HCl compared with the Au-PEI@NIST (Figure S7).

For further comparison, stability studies were conducted in additional biological media, including IMDM, EMEM, and F12, using UV–Vis (Figures S8 and S9) and DLS measurements (Table S2).

Finally, the thermal stability of the Au-NP suspensions was evaluated by UV–Vis absorbance measurements from 20 °C to 70 °C, which includes the pertinent range for most biomedical applications. Our samples were incubated at each temperature for 30 min prior to the absorbance measurements. All the AuNPs displayed a constant SPR band with respect to the temperature changes over the selected range (Figure S10), suggesting an adequate thermal stability, regardless of the surface polymer type and nanoparticle origin.

### 3.3. In Vitro Toxicity of Au-PEI and Au-PEG-COOH NPs

**Figure 2:**
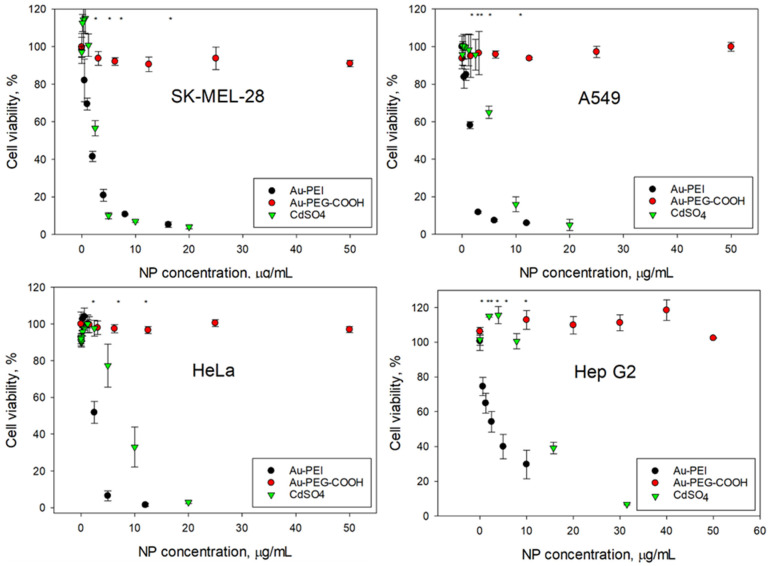
Cellular viability of cancerous cells (clockwise): SK-MEL-28, A549, Hep G2, and HeLa following 24 h exposure to Au-PEI@NIST and Au-PEG-COOH@NIST nanoparticles and CdSO4. * indicates *p* < 0.05.

**Figure 3:**
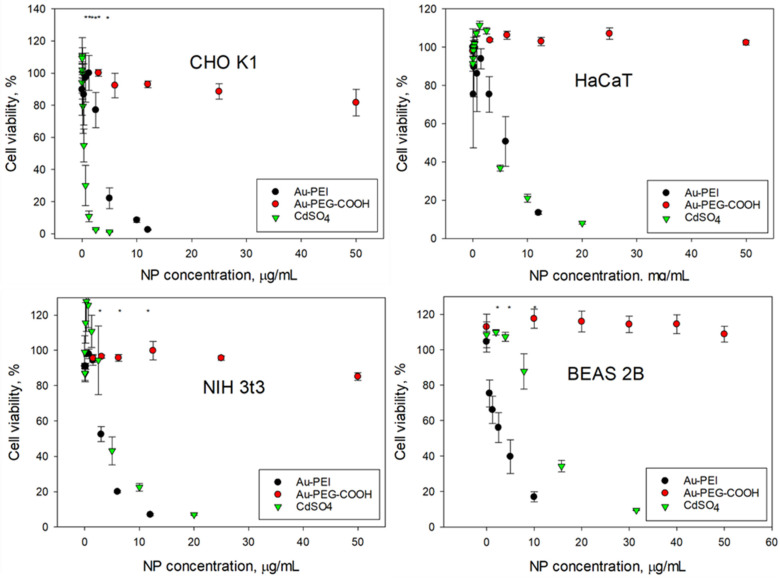
Cellular viability of normal cell lines (clockwise): CHO K1, HaCaT, BEAS 2B, and NIH3t3 following 24 h exposure to Au-PEI@NIST and Au-PEG-COOH@NIST nanoparticles and CdSO4. * indicates *p* < 0.05.

The AuNPs@NIST toxicity was tested with four cancer (SK-MEL-28, HeLa, A549, Hep G2) and four non-cancer (CHO K1, HaCaT, NIH 3t3, BEAS-2B) mammalian cell lines, representing various organs—skin, epithelium, and respiratory and digestive tracts. Figure 2 and Figure 3 show the cellular viability data obtained using an MTS assay following 24 h exposure to the Au-PEI@NIST, Au-PEG-COOH@NIST, and CdSO_4_ (as a chemical control). In all instances, the cellular viability dropped rapidly upon the increase of the Au-PEI@NIST concentration, with the IC_50_ in the range from 1.49 μg/mL to 4.7 μg/mL for these cell lines. The chemical control CdSO_4_ demonstrated a comparable toxicity, although generally lower than the Au-PEI@NIST, except for the CHO K1 cells (Figure 3) when the concentration was expressed in mass-based units. The cell viability did not decrease below 80% when exposed to the carboxylatedpolyethylene-glycol-terminated AuNPs (Au-PEG-COOH@NIST) at up to 50 μg/mL.

The toxicity of commercially sourced gold nanoparticles was measured with SK-MEL-28 (Melanoma) cells and is provided in Figure S11 for comparison purposes.

### 3.4. Effect of Free PEI Ligand

**Figure 4:**
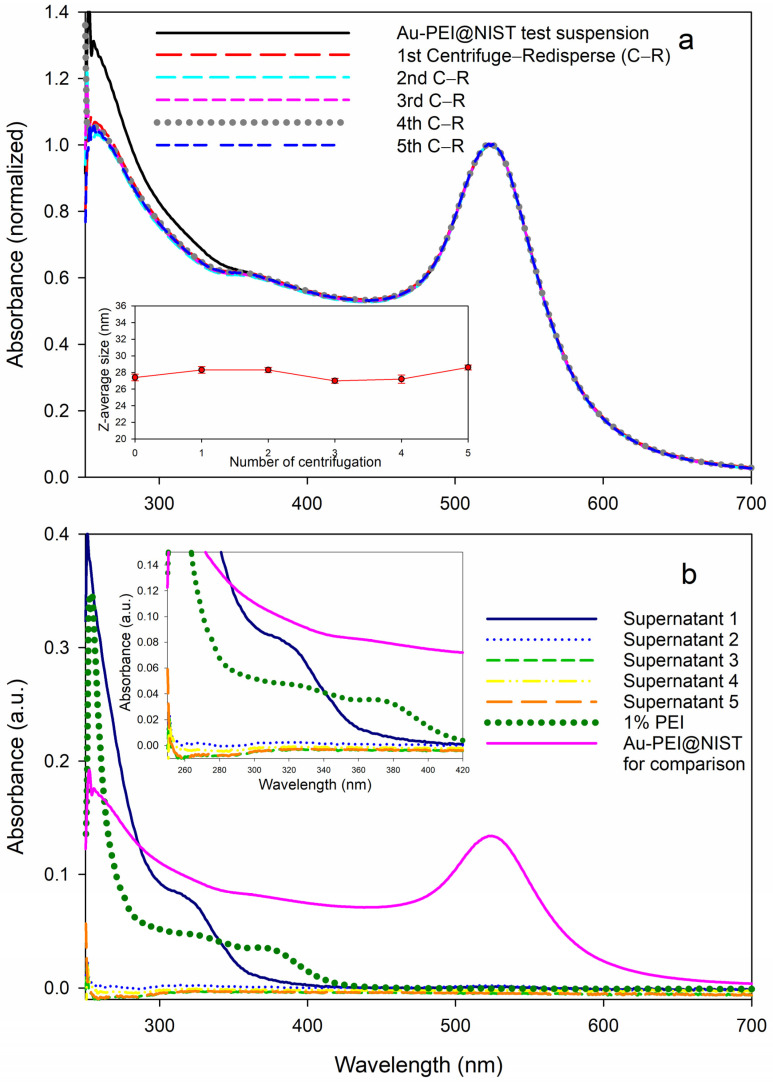
PEI release test of the Au-PEI@NIST via a series of centrifugal filtrations (CFs): UV–Vis measurements of (**a**) test suspension and CF-redispersed Au-PEI@NIST (inset: z-average sizes by DLS measurements as a function of CF number) and (**b**) CF-associated supernatants.

The UV–Vis measurements of the Au-PEI@NIST suspensions subjected to multiple centrifugal filtration cycles showed identical gold SPR bands regardless of the centrifugation cycle number (Figure 4a). The absorbance in the range 250 nm to 350 nm shown by the test suspension Au-PEI@NIST slightly decreased after the first centrifugal filtration, then it stabilized after the second through fifth cycles. This implies that some of the PEI was removed during the first filtration cycle (Figure 4b). Still, the particle size of the test suspension and redispersed Au-PEI@NIST was basically unchanged, as indicated by the DLS measurements (inset in Figure 4a), with the z-average particle diameter remaining close to 28 nm throughout the five CF cycles. If some of the PEI was released from the Au-PEI@NIST shell, it most likely was not covalently bound, but rather intercalated in the particle shell.

We measured the UV–Vis absorbance of the supernatants produced during the CF process. As shown in Figure 4b, the spectrum of the supernatant after the first CF shows a shoulder between 300 nm and 350 nm and further absorbance below 300 nm, which corresponded to the elevated absorbance of the test suspension of the Au-PEI@NIST in the same range (Figure 4a). Interestingly, the solution of pure 1% PEI exhibited an extra shoulder at 380 nm; however, no such shoulders appeared in the Au-PEI@NIST test suspension or supernatant 1 spectrum. This could be attributed to some type of chemical transformation of PEI during the reduction for the Au-PEI@NIST preparation, as proposed in previous reports [37,43]. Following four further centrifugation cycles, the Au-PEI@NIST NPs did not display meaningful absorbances in this range (Figure 4b), which confirmed no free PEI ligand.

**Figure 5:**
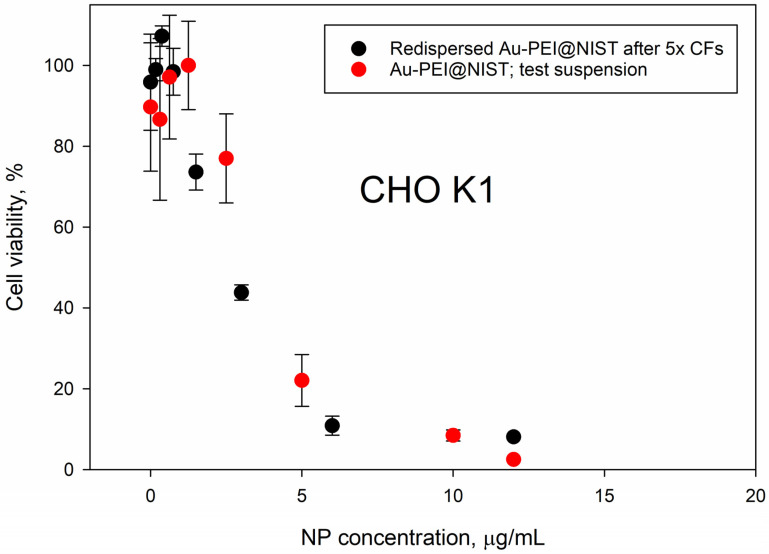
Cellular viability of CHO K1 cells, following 24 h exposure to Au-PEI@NIST that were redispersed in IMDM after 5× centrifugal filtration (black dots) and Au-PEI@NIST test suspension that were not subjected to centrifugation (red dots).

Figure 5 compares the toxicity data for the Au-PEI@NIST NPs prior to (red dots) and after (black dots) the reconstitution following 5× centrifugal filtration, recorded with CHO K1 cells. Although the PEI shell was partially released due to severe centrifugal treatments, as evident from the UV absorbance (Figure 4), the dose–response profiles for the CHO K1 cells were similar. This suggests that the biological activity of the Au-PEI@NIST remained unchanged, even with a marginal PEI coating loss. The filtrate toxicity data are presented in Figure S12.

### 3.5. Interference with MTS Assay

**Figure 6:**
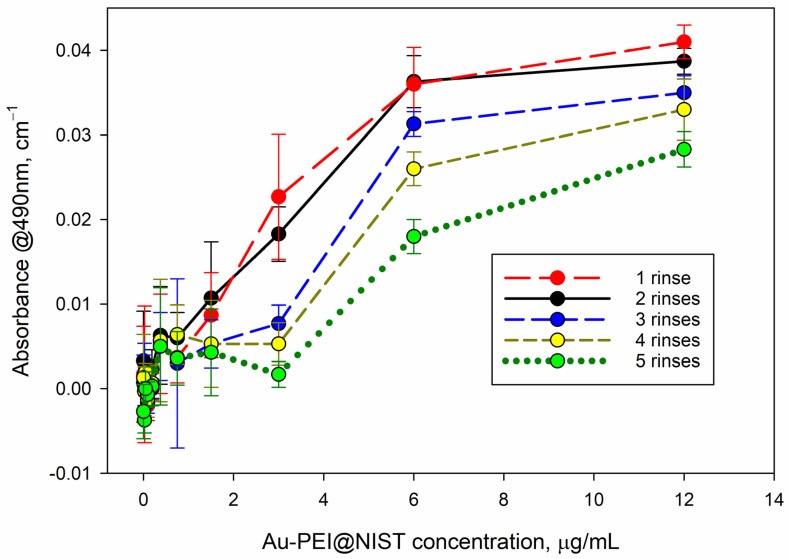
Residual absorbance of the plate following the decanting of the Au-PEI@NIST test suspension and repeated rinsing with 37 °C PBS. The Au-PEI@NISTs were incubated for 24 h with CHO K1 cells, grown to confluence.

AuNPs absorb light at the readout wavelength of the MTS assay (λ = 490 nm); therefore, they may potentially prejudice the assay readout if they are not completely removed from the plate wells prior to the MTS absorbance readout. We tested the residual absorbance of the AuNPs at λ = 490 nm after several rinses with warm PBS (Figure 6). As apparent from this graph, the Au-PEI nanoparticles were not fully removed from the higher concentration incubations (6 μg/mL–12 μg/mL), which resulted in about 0.02 OD–0.04 OD (optical density) residual readings at the MTS wavelength. Although this value is small (up to 5% of the typical formazan absorbance values in such tests), it may alter the cell viability estimate and should be evaluated before introducing the MTS reagent and subtracted from the final MTS absorbance readout. Such an optical interference could be circumvented if assays with an absorbance readout at the gold optical transparence range (λ > 650 nm) or based on luminescence (e.g., ATP) were utilized as alternatives to MTS. No residual absorbance was detected with the AuPEGCOOH NPs following the rinse with warm PBS.

## 4. Discussion

Given the applications of in vitro assays for MNM hazard screening are growing, we explored the potential of two types of AuNPs as cell viability assay controls. Earlier, it was determined that nanoparticle electrostatic charge is a major factor governing AuNP toxicity [31,32,33,38]. We compared the cytotoxicity of a positively charged Au-PEI and negatively charged Au-PEG-COOH NPs of similar size to eight cell lines, along with the extensive characterization of their physicochemical properties. The interest in benchmark materials for in vitro assays is driven by a significant scatter in the published assay data, encouraging the development of standardized protocols. Three general requirements were considered [26] for positive control materials to be applicable in cell viability assays—a consistent performance, availability, and safety. Consistent performance is directly related to minimizing the data variability when performing repeated tests in the same or different laboratories. It is achieved when the material is well characterized, stable, pure, and its preparation is not too complicated. In contrast to chemical compounds, the biological activity of particulate nanomaterials is highly dependent on their physicochemical properties, not just their composition and concentration. Positive control biological activity should be based on a similar mechanism of action as that of the assay that is used. The toxicity of AuNPs was extensively studied, and it is known to be highly dependent on the particle surface functionalization and particle size, and consequently, its effective surface area [33]. Our study did not address the mechanism of Au-PEI toxicity; however, it is well known that a positive particle charge facilitates favorable electrostatic interaction with a cellular membrane, leading to its disruption and/or MNM uptake [31,32,33]. Therefore, our working hypothesis is that a difference in surface charge polarity is the foremost reason for the in vitro toxicity contrast between Au-PEI@NIST and Au-PEG-COOH@NIST. We also have evidence that a lower positive Au-PEI particle charge from some commercial sources (Au-PEI@NIST, Au-PEI@C1, and Au-PEI@C2) results in their lower cytotoxicity when tested with SK-MEL-28 cells (Table S2, Figure S11). A detailed examination of the biological activity mechanism was beyond the scope of this work and could be addressed in subsequent studies.

Nanoparticle comprehensive characterization is a labor-intensive process, requiring specialized equipment and expertise that is not widely available in laboratories that specialize in biological testing. Our test materials, Au-PEI@NIST and Au-PEG-COOH@NIST nanoparticles, were synthesized using previously published detailed procedures [36,37,38,39,42] and were characterized in terms of their particle size (TEM and DLS), surface charge (ZP), and colloidal stability, and tested over a wide range of temperatures, solution compositions, and acidity. The particles could be easily dispersed in PBS and several types of cell culture media with and without FBS. When dispersed in cell culture media, the Au-PEI@NIST slightly agglomerated over a 48 h period (Figure S4) and were stable over the wide range of pH. In contrast, the Au-PEG-COOH@NIST were less stable than the Au-PEI@NIST in culture media and in extremely acidic and alkaline environments (Figure S5). When dispersed in DIW, the materials could be stored for up to at least 6 months (Au-PEG-COOH@NIST) or three years (Au-PEI@NIST) at room temperature (Figure 1). The colloidal stability over the wide range of suspension conditions suggests the control material’s applicability in different experimental setups. We also tested the robustness of the gold nanoparticle functionalization by subjecting these materials to several cycles of centrifugation and filtration (Figure 4). As suggested by the absorbance in the UV range (Figure 4), some of the PEI ligand could be present as a leftover from the synthesis or released from the Au-PEI@NIST NPs after these treatments, yet with an inconsequential effect on the NP cytotoxic activity (Figure 5). This confirmed the inherent Au-PEI@NIST toxicity, which was not disguised by the presence of free PEI in the solution. The 24 h CHO K1 incubations with the AuPEI@NIST filtrate in DIW showed an up to 40% drop in cell viability, regardless of the centrifugation cycle number; however, part of this drop could be attributed to media depletion effects in the higher filtrate fractions (Figure S12).

Consistent performance of the control material also includes the generation of the full dynamic range for the assay without interference with the assay readout. Our AuNPs@NIST performance in the cell viability tests was established with the MTS assay in four cancer and four normal mammalian cell lines from various organs. As shown in Figure 2 and Figure 3, the cellular viability steadily decreased from no response to complete inhibition when the Au-PEI@NIST (potential positive control) concentration was increased to 12 μg/mL, while staying above 80% when incubated for 24 h with the Au-PEG-COOH@NIST, a candidate negative control. The IC_50_ for the Au-PEI@NIST was in the range from 1.49 μg/mL to 4.7 μg/mL for the eight tested cell lines. In many nanotoxicity studies, the nanoparticle dose is quantified using the particle number and exposed surface area units; therefore, we provide the IC_50_ for the cells exposed to Au-PEI@NIST in these units (Table 2). For comparison purposes, our cells were also exposed to a widely used chemical toxin CdSO_4_, a positive control [41]. As evident from Figure 2 and Figure 3, the cell viability repeatedly increased above 100% when exposed to low concentrations (up to 3 μg/mL) of this toxin, indicating growth stimulation due to a possible hormesis effect [44]. This phenomenon was more pronounced for certain cell types (Figure 2 and Figure 3) and was a confounding factor in the acquisition of the full dose–response profile. In contrast, cell incubations with the Au-PEI@NIST in the same concentration range did not induce growth stimulation, while consistently producing a cell viability decay.

AuNPs exhibit UV–Vis absorbance due to free electron plasma in the spectral range λ < 650 nm, which may interfere with the MTS readout at λ = 490 nm. We found that the Au-PEI@NIST tended to adhere to the cell and/or incubation plate (plastic) surface and were not fully removed, even after several rinse cycles in warm PBS (Figure 6). This extra absorbance was not negligible; however, the final MTS optical density reading could be corrected by performing a control absorbance measurement prior to introducing the MTS reagent and subtracting the interfering absorbance from the recorded value. Alternatively, assays with absorbance readouts in the gold transparency range that are based on light emission or non-optical sensing (e.g., electrochemical impedance) should be free of this issue. An interlaboratory study that includes several viability assay platforms would reveal and validate the optimal experimental setup for application of these potential control materials.

Commercial availability of the control material is recognized as key to its accessibility to assay users. Ideally, they should be able to obtain them from multiple vendors in case production is discontinued by one company. Various types of gold nanoparticles are currently available commercially, including those functionalized with PEI and PEG-COOH. We identified at least two commercial sources of these particles—Nanopartz. Inc. and nanoComposix, Inc.—that demonstrated comparable toxicity, as tested with Sk-MEL-28 cells. However, it is not realistic to expect identical physicochemical and biological activity properties from materials that were prepared using unpublished proprietary procedures. Therefore, reference materials prepared under an established quality control system by a reputable metrology laboratory would be preferable as assay controls. Finally, control material safety for the user and easy disposability have been listed as important characteristics. Both Au-PEI and Au-PEG-COOH NPs are non-volatile, but they are toxic if inhaled or ingested; therefore, standard protective measures as required for work with nanomaterials should be applied during their use, even when the typical amount needed to perform in vitro nanotoxicology assays is small—quantified in micrograms. Aqueous suspensions of AuNPs are chemically inert and would not react with other compounds in the waste disposal collection. Their discarding is not strictly regulated as opposed to Cd compounds, which must conform to heavy metal waste disposal requirements.

## 5. Conclusions

The demand for reference materials in the hazard screening of nanomaterials is well documented. It derives from the development of in vitro and in silico substitutes to ensure the safe use of novel nanoproducts. In vitro cell viability measurements following a fixed period exposure of an individual cellular system to MNM have been commonly adopted as a first-tier tactic to anticipate the response of a living biological system. These assays produce toxicant dose–response profiles and can be performed using multi-sample platforms, combined with robotic arrangements. Still, a significant variability in biological assay outputs hampers the data comparison across laboratories, as well as test reproducibility. An analysis of assay variability factors have determined that the lack of standardization here is a major issue. A key element of a standard assay is benchmarking to an expected output value when an internal standard is tested alongside the material of interest. Ideally, a pair of such control materials would show the full dynamic range for the cell viability assay, from complete cell growth inhibition to full viability.

We explored the utility of AuNPs as positive and negative controls in cell viability assays. The nanoparticles had hydrodynamic diameters of approximately 30 nm and were functionalized with polyethylenimine (Au-PEI@NIST) and carboxylated polyethylene glycol (Au-PEG-COOH@NIST), which resulted in cationic and anionic charges, respectively. Both preparations were extensively characterized in terms of their size/size distribution, zeta potential, and mass ratio of PEI/Au, and their colloidal stability was tested in various physiological media, as well as a range of suspension temperatures and acidities. Stability measurements showed a shelf-life for up to three years in DIW at room temperature and at least 24 h in cell growth media. The acute toxicity of cationic charged gold nanoparticles was confirmed in eight mammalian cell lines representing various organs using dose–response measurements after 24 h incubations with the MTS assay. A full cell growth inhibition was recorded at *C*_AuPEI@NIST_ = 12 μg/mL, while the cell viability remained above 80% for *C*_AuPEGCOOH@NIST_ = 50 μg/mL for all the tested cell lines. We found that the AuPEI@NIST NPs were superior in comparison with CdSO_4_ as a positive nanotoxicology in vitro control, as they produced a more consistent dose–response profile that lacked cell growth stimulation at low concentrations. However, cationic charged NPs had a propensity to adhere to the cell and/or well plastic surface and were not fully removed after several PBS rinses. This resulted in optical interference with the MTS readout at 490 nm, which required additional measurements with subsequent corrections. In summary, we demonstrated the utility of Au-PEI@NIST and Au-PEG-COOH@NIST materials as in vitro controls for nano-hazard screening. A wider, interlaboratory study, that includes additional kinds of cell viability assays, would be essential to validate these controls as appropriate for reference materials.

## Figures and Tables

**Table 2:** Summary of IC_50_ for Au-PEI@NIST expressed in mass-, particle number-, and surface-area-based units.

Cell Type	IC_50,_ mg/mL	IC_50,_ Particles/mL ^a^	IC_50,_ cm^2^/mL ^b^
A549	1.76 ± 0.49	(1.14 ± 0.32) × 10^11^	2.36 ± 0.66
CHO K1	1.89 ± 0.28	(1.22 ± 0.18) × 10^11^	2.52 ± 0.37
HaCaT	4.70 ± 1.89	(3.11 ± 1.20) × 10^11^	6.44 ± 0.87
HeLa	2.21 ± 0.12	(1.4 ± 0.07) × 10^11^	2.90 ± 0.14
SK-MEL-28	1.49 ± 0.32	(0.97 ± 0.19) × 10^11^	2.00 ± 0.41
NIH 3T3	2.79 ± 0.41	(1.82 ± 0.26) × 10^11^	3.76 ± 0.54
HepG2	3.95 ± 0.59	(2.56 ± 0.38) × 10^11^	5.31 ± 0.76
BEAS-2B	3.15 ± 0.47	(2.04 ± 0.31) × 10^11^	4.23 ± 0.63

Uncertainties indicate a 95% confidence interval from the data fitting to a sigmoidal function using SigmaPlot software. a—Particle number was estimated based on Au core diameter *D*_TEM_ = (11.5 ± 1.8) nm. b—Particle surface area was estimated using the z-average hydrodynamic diameter *D*_z_ = (25.7 ± 0.4) nm.

## Data Availability

Study data are available upon request from vytas@nist.gov and taejoon.cho@nist.gov.

## Supplementary Materials

The following supporting information can be downloaded from https://www.mdpi.com/article/10.3390/nano15020079/s1, Table S1: TEM data; Table S2: Nominal values of sizes, zeta potential, SPR, and pH of AuNPs in biological media; Figure S1: Violin plot of particle diameter data; Figure S2: Sample images from Au PEI@NIST sample. Scale bars are equal to 30 nm and 3 nm; Figure S3: Example images from the Au-PEG-COOH@NIST sample. Scale bars are equal to 30 nm and 3 nm; Figure S4: Representative data showing the colloidal stability of Au-PEI@NIST and Au-PEG-COOH@NIST over 48 h at 20 °C evaluated in physiological media: by UV-Vis (a) Au-PEI@NIST in PBS, (b) Au-PEG-COOH in PBS, (c) Au-PEI@NIST in DMEM, (d) Au-PEG-COOH@NIST in DMEM, (e) Au-PEI@NIST in 10 % FBS-DMEM, (f) Au-PEG-COOH@NIST in 10 % FBS-DMEM; Figure S5: Colloidal stability of AuNPs@NIST over the pH range (1 to 12) measured over 24 h. Colloidal stability of AuNP suspensions was assessed at different acidity levels. As already shown for PBS, all AuNPs are stable at neutral pH, therefore colloidal behavior of Au-PEI@NIST was evaluated in 50 mmol/L HCl (pH 1.2) and 50 mmol/L NaOH (pH 12) to cover the maximum possible pH range of interest. (Figure S5a–c). While Au-PEG-COOH@NIST (Figure S5d–f) was destabilized in extremely acidic or basic solutions; Figure S6: Representative data showing the colloidal stability of Au-PEI@Cs and Au-PEG-COOH@C1 over 48 h at 20 °C evaluated in physiological media: by UV-Vis (a) Au-PEI@C1 in PBS, (b) Au-PEI@C2 in PBS, (c) Au-PEG-COOH@C1 in PBS, (d) Au-PEI@C1 in DMEM, (e) Au-PEI@C1 in DMEM, (f) Au-PEG-COOH@C1 in DMEM, (g) Au-PEI@C1 in 10 % FBS-DMEM, (h) Au-PEI@C2 in 10 % FBS-DMEM, (i) Au-PEG-COOH@C1 in 10 % FBS-DMEM; Figure S7: Colloidal stability of commercially sourced AuNPs, exposed for 24 h to solutions with different acidity over the pH range (1 to 12); Figure S8: Colloidal behavior of Au-PEI@NIST and Au-PEG-COOH@NIST in (a,b) IMDM, (c,d) in EMEM, and (e,f) in F12; Figure S9: Colloidal behavior of Au-PEI@NIST and Au-PEG-COOH@NIST in (a,b) IMDM, (c,d) in EMEM, and (e,f) in F12; Figure S10: Thermal stability of all tested AuNPs over the temperature range from 20 °C to 70 °C; Figure S11: Cellular viability of skin cancer cells SK-MEL-28 following 24 h exposure to Au-PEI and Au-PEG-COOH nanoparticles from two commercial sources C1 and C2 overlayed with Au-PEI@NIST data; Figure S12: Toxicity of AuPEI@NIST filtrate tested with CHO K1 cells at three concentration levels–stock (1.0), 50% and 25% dilution with full growth media (0.5 and 0.25).
