# Supplementary material for: Well-Characterized Polyethyleneimine-/Carboxylated-Polyethylene-Glycol-Functionalized Gold Nanoparticles as Prospective Nanoscale Control Materials for In Vitro Cell Viability Assays: Particle Characterization and Toxicity Tests in Eight Mammalian Cell Lines
Source: Nanomaterials (Basel). 2025 Jan 7;15(2):79. doi: 10.3390/nano15020079 (PMC11767793; doi:10.3390/nano15020079)
Supplement: Supplementary file 1 [file nanomaterials-15-00079-s001.zip › nanomaterials-3390581-supplementary.pdf]

Supporting Information

Well characterized polyethyleneimine/carboxylated polyethylene glycol functionalized gold nanoparticles as prospective nanoscale control materials for *in vitro* cell viability assays: particle characterization and toxicity test in eight mammalian cell lines

Vytas Reipa[a], Vincent A. Hackley[a], Alessandro Tona[a], Min Beom Heo[b], Ye Ryeong Lee[b], Tae Geol Lee[b], Aaron Johnston-Peck[a], and Tae Joon Cho[a]

[a]Materials Measurement Laboratory, National Institute of Standards and Technology, Gaithersburg, Maryland, USA

[b]Safety Measurement Institute, Korea Research Institute of Standards and Science, Daejeon, Korea

Measurement methods and instrumentation for physico-chemical properties

UV-Vis spectra were collected using a Perkin Elmer Lambda 750 spectrophotometer (Waltham, MA, USA) equipped with an 8+8 cell changer and water-jacketed temperature control. Dynamic light scattering (DLS) was performed using a Zetasizer (Nano ZS, Malvern Instruments, Westborough, MA, USA) operated in backscatter mode at 173° with a laser wavelength of 633 nm. Batch-mode DLS measurements followed the NIST-NCL (Nanotechnology Characterization Laboratory) Protocol PCC-1 _with a z-average diameter reported as the mean of at least five measurements ± one standard deviation. Zeta potential measurements were obtained with Nano ZS using the palladium electrodes mounted in a dip cell and applying the Smoluchowski equation for thin double layers, reported as the mean of no less than three measurements ± one standard deviation. UV-vis absorbance spectra were obtained using disposable plastic semi-micro cuvettes (Brandtech, Inc., Essex, CT, USA) with a 1 cm path length. All DLS, zeta potential, and UV-vis measurements were conducted at (20 ± 0.1) °C unless noted otherwise. The surface coverage was assessed by performing thermogravimetric analysis (TGA) using the STA 449 F1 Jupiter instrument (NETZSCH, Burlington, MA).

TEM measurements

A drop of particles suspended in water were placed on a carbon film supported by a copper grid and dried. Samples were imaged using a Thermo Fisher Scientific (formerly FEI) Titan 80-300 transmission electron microscope, operating at an accelerating voltage of 300 kV. After alignment, the objective lens current remained fixed and focusing was conducted by adjusting the sample height. The pixel size was calibrated using the lattice spacings of the Au particles as an internal reference. The lattice parameter of the particles was assumed to be 0.4078 nm. An FFT (fast Fourier Transform) of an image was calculated and a circle was manually fit to the {111} set of reflections to derive the pixel size. This was repeated on four images and the mean value was used.

Data was analyzed and processed using various tools embedded in Fiji software. The images were processed by applying a Gaussian blur with a sigma of 2 pixels and then images were binned from the original size of (4096 x 4096) pixels to format of (2048 x 2048) pixels. A threshold, using "Auto Threshold v.1.80.0" with either the "Maximum Entropy" or "Percentile" algorithm, was applied converting the grayscale image into a binary representation and any holes were closed. The particle area was measured using the "Analyze Particles" function, limiting results to particles with a circularity greater than 0.5 to eliminate particles from the analysis where the fidelity of the thresholding was poor. Particle diameter was calculated using the formula for the area of a circle. The reported uncertainty is one standard deviation about the mean.

Table S1.

Sample	Mean Diameter (nm)	Standard Deviation (nm)	n
Au-PEI@NIST	11.5	1.8	739
Au-PEG-COOH@NIST	14.3	0.9	982

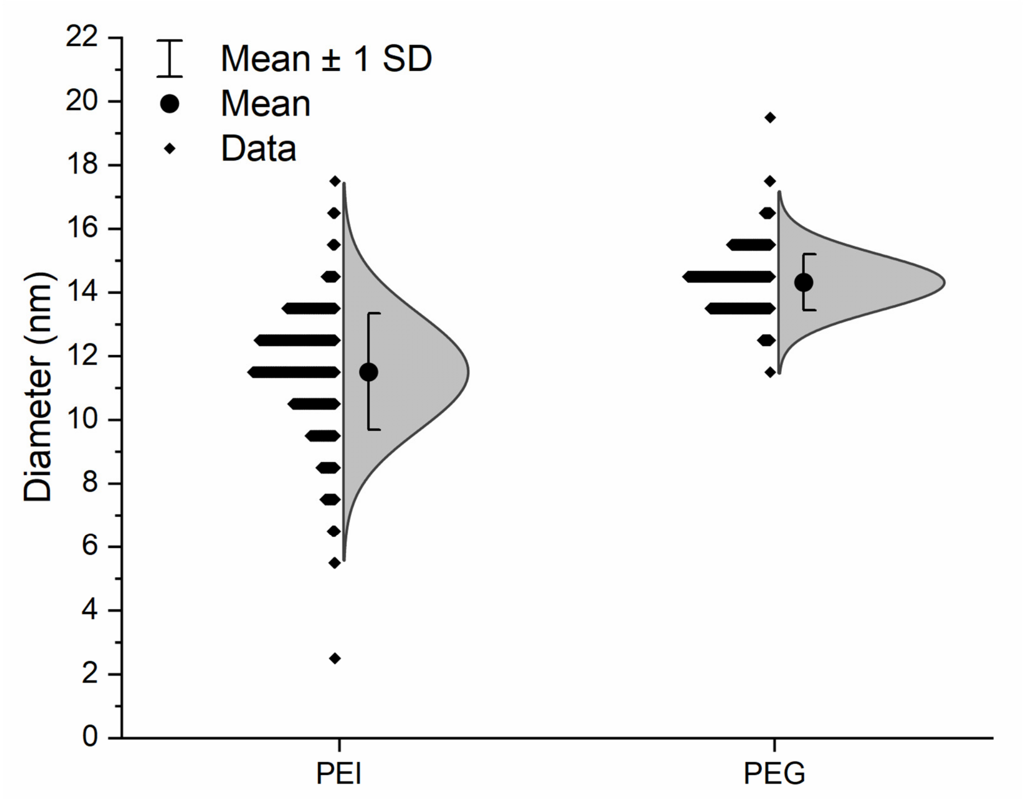

Figure S1. Violin plot of particle diameter data

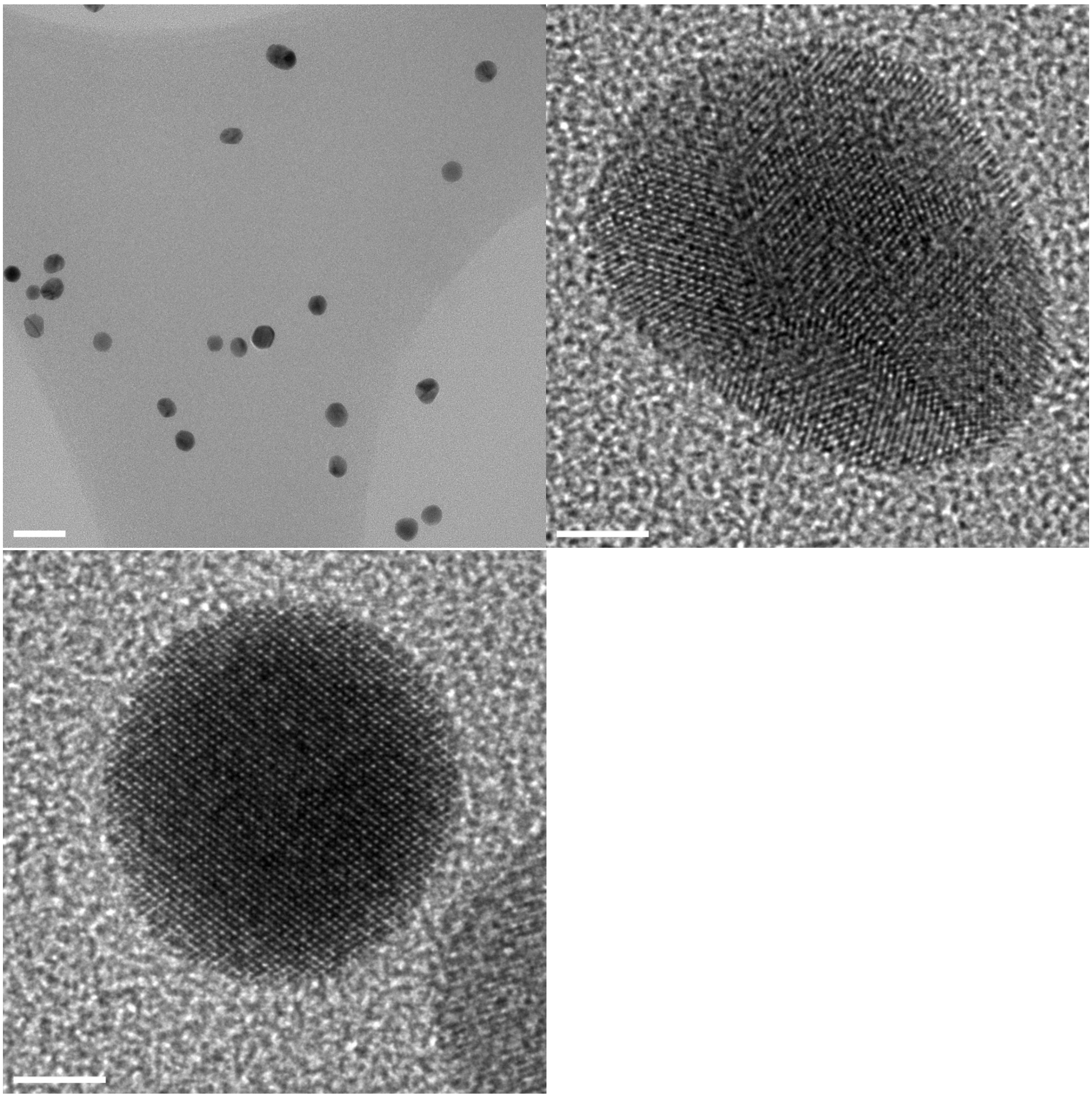

Figure S2. Sample images from Au PEI@NIST sample. Scale bars are equal to 30 nm and 3 nm.

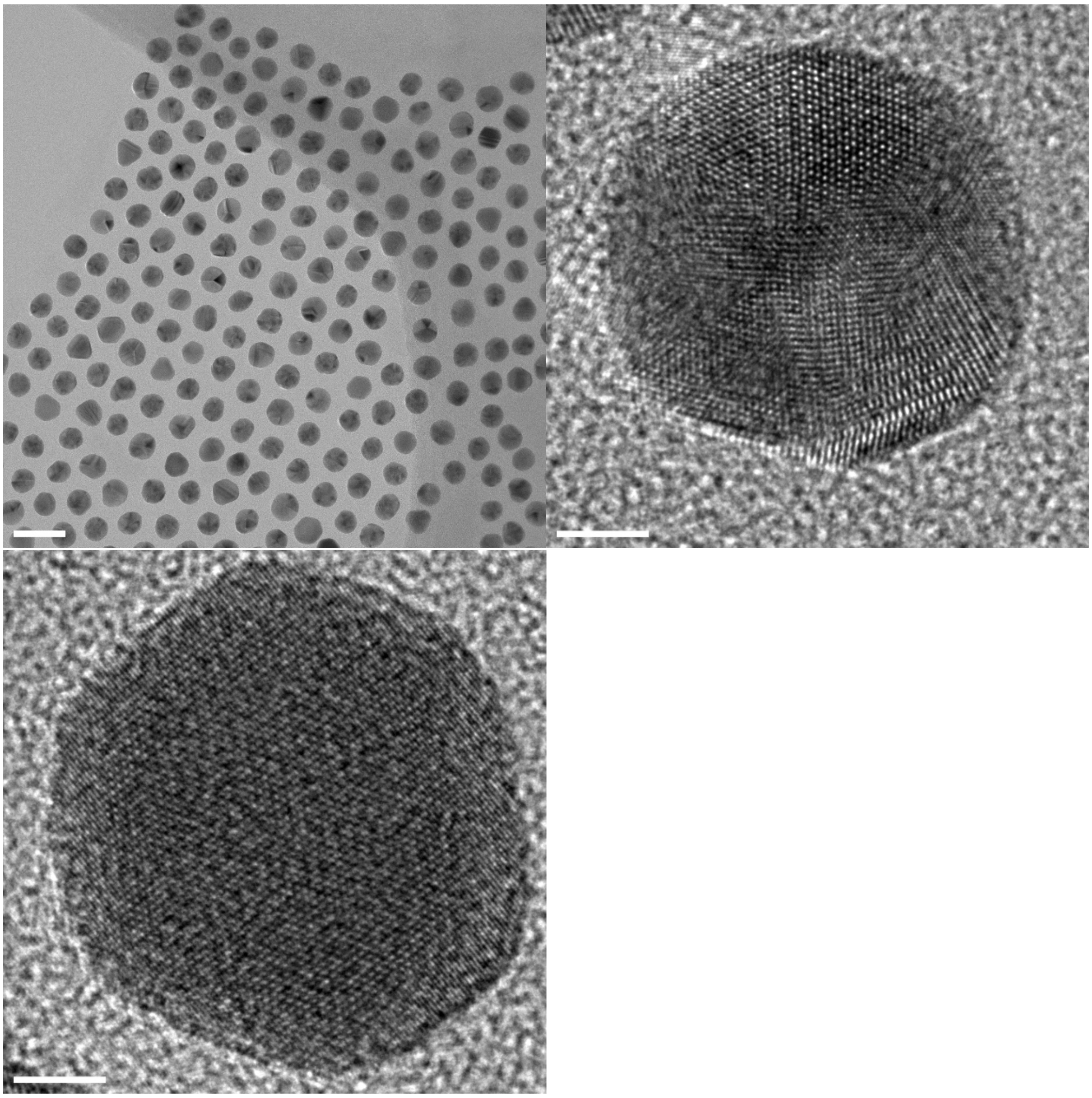

Figure S3. Example images from the Au-PEG-COOH@NIST sample. Scale bars are equal to 30 nm and 3 nm.

Stability test

Shelf-life: Each Au-PEI@NIST and Au-PEG-COOH@NIST was contained in a glass bottle with a cap and stored at room temperature (ambient condition) without any aid of excipients or aging acceleration process for at least 6 months, then assessed by DLS and UV-Vis and comparison with the fresh sample measurements prepared was made while maintaining identical measurement conditions.

Stability In physiological media: Stability in physiological media should be tested over 48 h period as it is a relevant timescale to many cell-based exposure assays. Test materials were dispersed in a selected medium and evaluated immediately. After the first measurement, a measurement was performed every '*n*' hours over 48 h with at fixed concentration and instrumental set-ups.

Interpretation of stability test results:

Interpretation of stability test results is a discussion of the behavioral changes of the test materials before and after based on measurement results and observations. DLS measurements provide information on the change of the hydrodynamic size and/or size distribution (modality) of the test materials. Increased size after a test indicates that the test materials have agglomerated or aggregated. A broadened size distribution (occasionally with multi-modality) also indicates the perturbating factor (time, temperature, pH, matrix, etc.) has resulted in morphological transformation.

Moreover, a change in the SPR band measured by UV-Vis absorbance is another indicator of the colloidal stability. The red-shifted SPR band for the test material after exposure to a certain test condition indicates the occurrence of a size increasing event. Broadening of the SPR band relative to the initial spectrum is also evidence of a broadening size distribution. Additionally, if

the optical density of the SPR peak is reduced over time without an associated red-shift, this observation suggests removal of the test material from the detection zone, probably as a result of agglomeration/aggregation followed by rapid sedimentation or coating on the side walls of the cuvette (i.e., loss of material to the cell) during measurements. This observation should be validated by comparison with DLS results on the same material.

Fig. S4 shows the colloidal behavior of Au-PEI@NIST in physiological media. The SPR bands of each Au-PEI@NIST and Au-PEG-COOH@NIST exhibited identical spectral shape over 48 h in PBS buffer (Fig. S4a, b), demonstrating sufficient resistance to the charge screening effect that could lead to agglomeration of charge-stabilized particles due to elevated ionic strength. Dispersion in DMEM, a common cell culture medium that includes biological constituents in addition to salts, resulted in marginal particle destabilization. Au-PEI@NIST exhibited a ≈ 5 % decrease in the SPR maximum intensity and a red-shift ($\Delta\lambda_{max}$ ≈ 7 nm) in UV-Vis spectra over 48 h in DMEM (Fig. S4c). It was observed that between 36h and 48 h, absorbance increased near a wavelength of 600 nm (crossover at ≈ 550 nm), which suggests particle agglomeration as subsequently confirmed by DLS data (Table S2 in Supplemental Information). This is likely caused by interaction with DMEM biological components. On the other hand, Au-PEG-COOH@NIST showed a significant decrease in absorbance (≈ 32 %) during the first 6 h in DMEM, but no discernible red-shifting of the plasmon peak after 48 h (Fig. 2d). We also observed particle deposition on the wall of the UV-Vis cuvette, which is consistent with the drastic SPR peak decrease in DMEM over time reflecting the reduction in solution particle concentration.

Stability tests were conducted in DMEM supplemented with 10 % FBS (Fig. S4e, f). There, the stability of AuNPs@NIST colloids was lower compared to PBS, but above that measured in DMEM without FBS. This suggests that stability is aided by the protein corona formed in FBS, that provides an additional shield protecting the AuNPs from interactions with destabilizing DMEM ingredients.

Table S2. Nominal values of sizes, zeta potential, SPR, and pH of AuNPs in biological media

AuNPs	***Medium***	***ZP* (mV)/*pH***	***SPR* (nm)[a] 0h→48 h**	**D_z (nm)[a] 0h→48 h**
Au-PEI@NIST	PBS	+1.1 ± 0.1/9.5	522→523	26.0→25.9
	DMEM	+5.4 ± 3.2/8.6	522→529	26.0→163.9
	FBS/DMEM	-4.3 ± 0.9/8.8	525→525	26.3→129.8
	IMDM	+13.3 ± 1.6/8.0	522→528	32.5→28.5
	EMEM	+16.2 ± 1.3/9.1	523→527, 578	36.9→259.6
	F12	-2.7 ± 0.5/8.8	523→527	26.4→71.6
Au-PEG-COOH@NIST	PBS	-5.2 ± 2.0/7.7	522→523	31.2→32.7
	DMEM	-5.0 ± 0.8/8.3	522→523	32.7→144.3
	FBS/DMEM	-7.4 ± 0.7/8.5	521→521	25.6→129.7
	IMDM	-2.9 ± 0.6/7.6	522→528	33.9→93.1
	EMEM	-4.4 ± 1.6/8.7	523→523, 580	32.6→46.8
	F12	-7.2 ± 0.5/8.3	521→522	26.0→71.1
Au-PEI@C1	PBS	-11.5 ± 0.8/11.0	520→520	19.7→20.5
	DMEM	-7.5 ± 0.4/10.8	519→ppt'ed	86.5→ppt'ed
	FBS/DMEM	-12.5 ± 0.5/10.2	530→535[b]	259.4→263.8[b]
	IMDM	-3.6 ± 0.3/10.5	520→ppt'ed	113.9→ppt'ed
	EMEM	-11.3 ± 1.8/10.9	507→ppt'ed	1150→ppt'ed
	F12	-14.0 ± 0.1/10.7	521→522, 585	173.2→216.3
Au-PEI@C2	PBS	+12.1 ± 1.9/7.7	525→525	24.9→25.1
	DMEM	+4.6 ± 2.4/8.2	526→527	32.1→ppt'ed
	FBS/DMEM	-9.3 ± 1.2/8.4	531→526[b]	234.9→174.6[b]

	IMDM	+13.6 ± 3.0/7.9	525→529	29.9→28.4
	EMEM	+4.6 ± 3.6/8.7	523→526	36.3→135.3
	F12	-10.6 ± 0.8/8.5	528→537	395→200
Au-PEG-COOH@C1	PBS	-10.2 ± 1.3/7.6	517→518	31.8→32.0
	DMEM	-9.8 ± 0.6/8.2	520→521	33.4→1160
	FBS/DMEM	-8.3 ± 0.7/8.4	523→526	25.1→54.6
	IMDM	-12.4 ± 0.7/7.7	519→520	33.8→29.3
	EMEM	-14.6 ± 2.4/8.7	520→521	32.7→45.3
	F12	-9.4 ± 1.2/8.3	517→519	24.0→29.2

a: Single measurement without any uncertainty. For trend observation only, *b*: Partially precipitated.

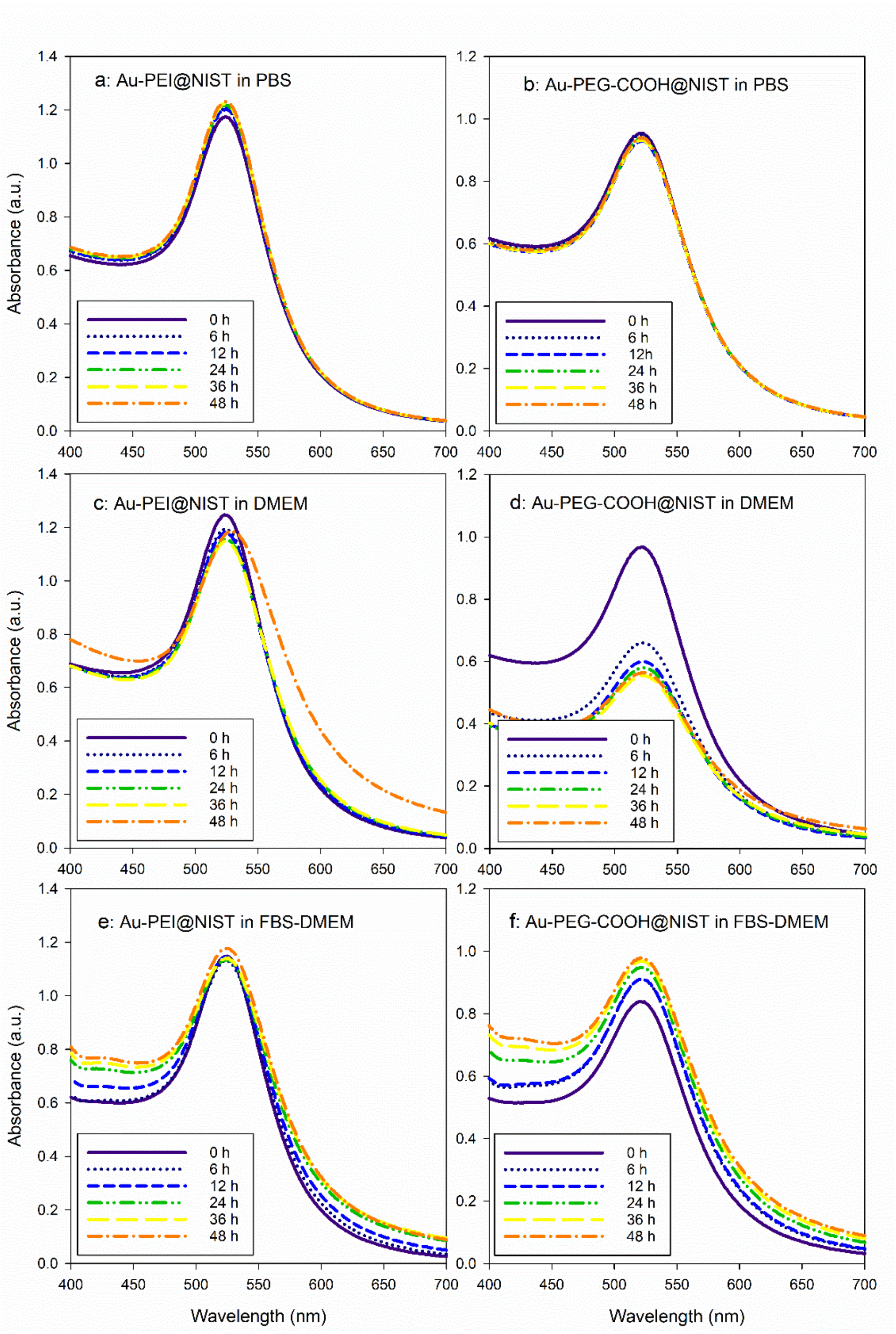

Figure S4. Representative data showing the colloidal stability of Au-PEI@NIST and Au-PEG-COOH@NIST over 48 h at 20 °C evaluated in physiological media: by UV-Vis (a) Au-PEI@NIST in PBS, (b) Au-PEG-COOH in PBS, (c) Au-PEI@NIST in DMEM, (d) Au-PEG-COOH@NIST in DMEM, (e) Au-PEI@NIST in 10 % FBS-DMEM, (f) Au-PEG-COOH@NIST in 10 % FBS-DMEM.

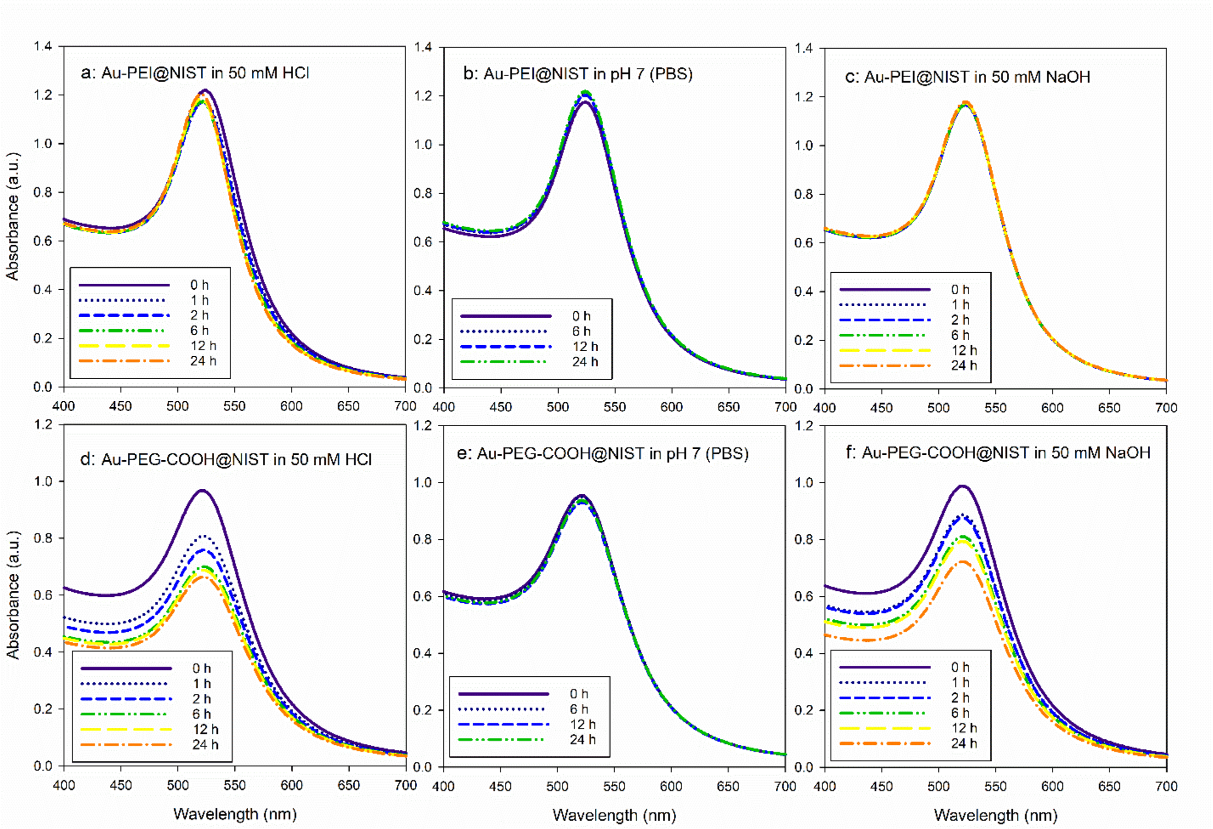

Figure S5. Colloidal stability of AuNPs@NIST over the pH range (1 to 12) measured over 24 h. Colloidal stability of AuNP suspensions was assessed at different acidity levels. As already shown for PBS, all AuNPs are stable at neutral pH, therefore colloidal behavior of Au-PEI@NIST was evaluated in 50 mmol/L HCl (pH 1.2) and 50 mmol/L NaOH (pH 12) to cover the maximum possible pH range of interest. (Figure S5a-c). While Au-PEG-COOH@NIST (Fig. S5d–f) was destabilized in extremely acidic or basic solutions.

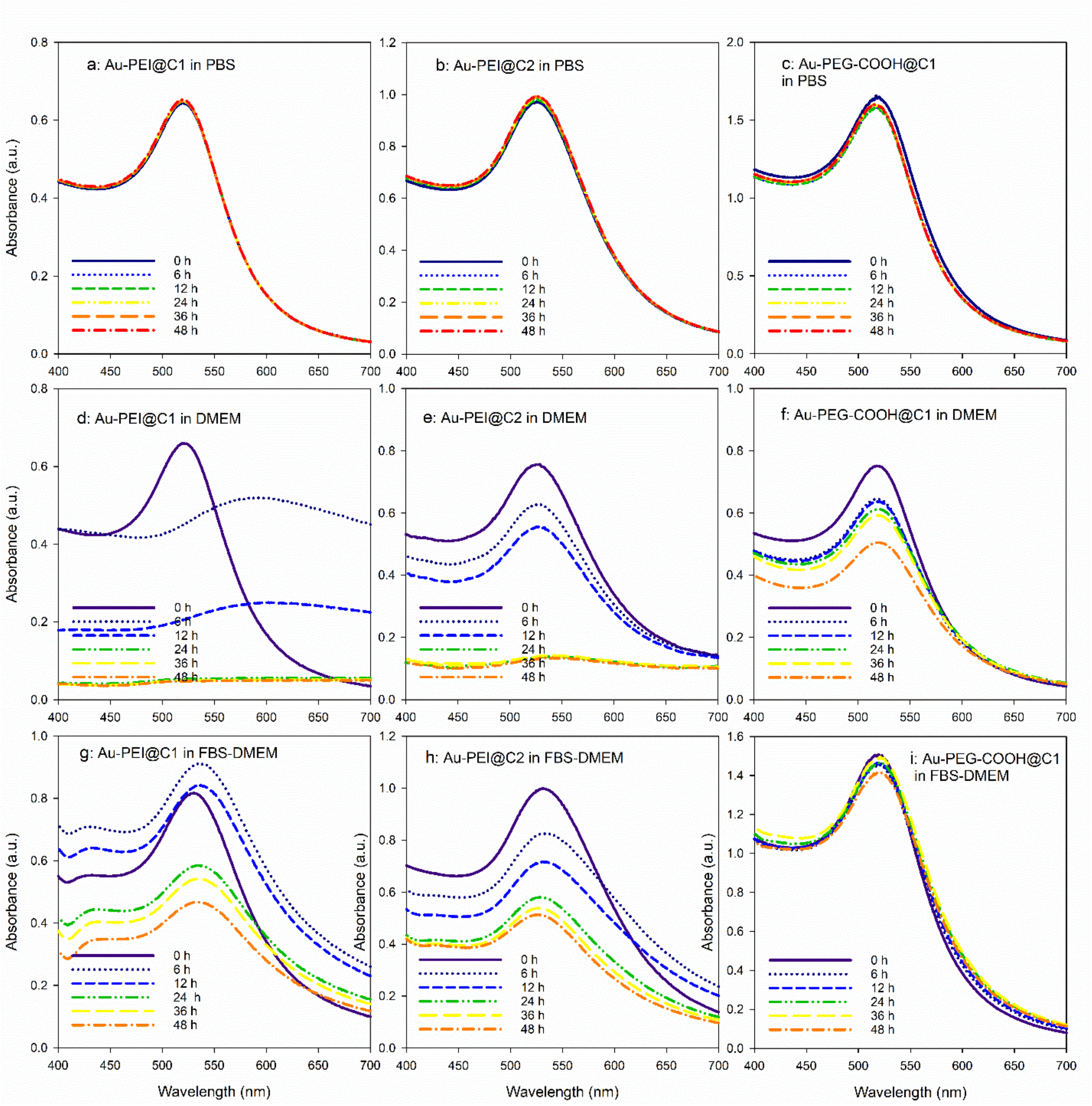

Figure S6. Representative data showing the colloidal stability of Au-PEI@Cs and Au-PEG-COOH@C1 over 48 h at 20 °C evaluated in physiological media: by UV-Vis (a) Au-PEI@C1 in PBS, (b) Au-PEI@C2 in PBS, (c) Au-PEG-COOH@C1 in PBS, (d) Au-PEI@C1 in DMEM, (e) Au-PEI@C1 in DMEM, (f) Au-PEG-COOH@C1 in DMEM, (g) Au-PEI@C1 in 10 % FBS-DMEM, (h) Au-PEI@C2 in 10 % FBS-DMEM, (i) Au-PEG-COOH@C1 in 10 % FBS-DMEM.

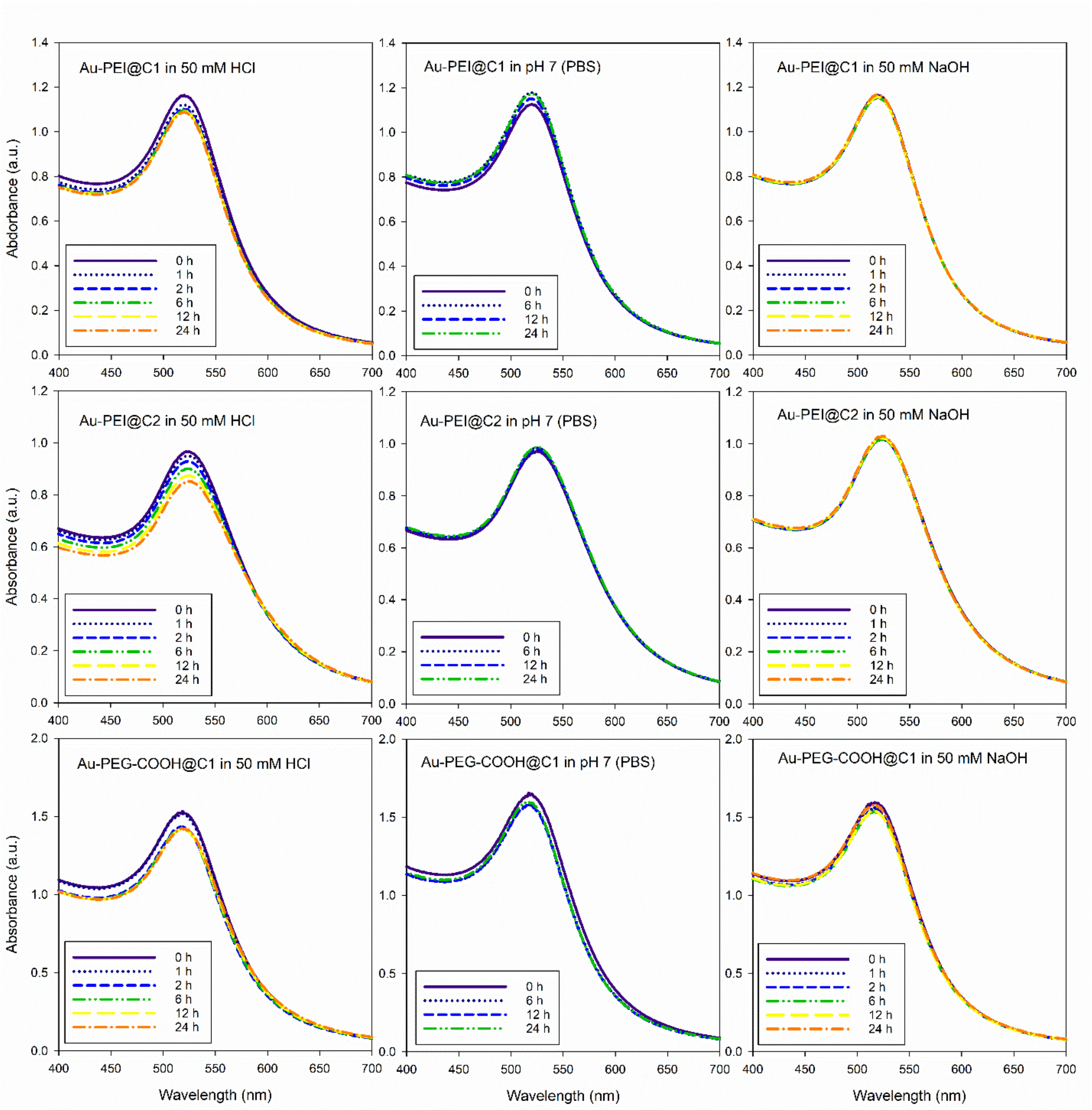

Figure S7. Colloidal stability of commercially sourced AuNPs, exposed for 24h to solutions with different acidity over the pH range (1 to 12).

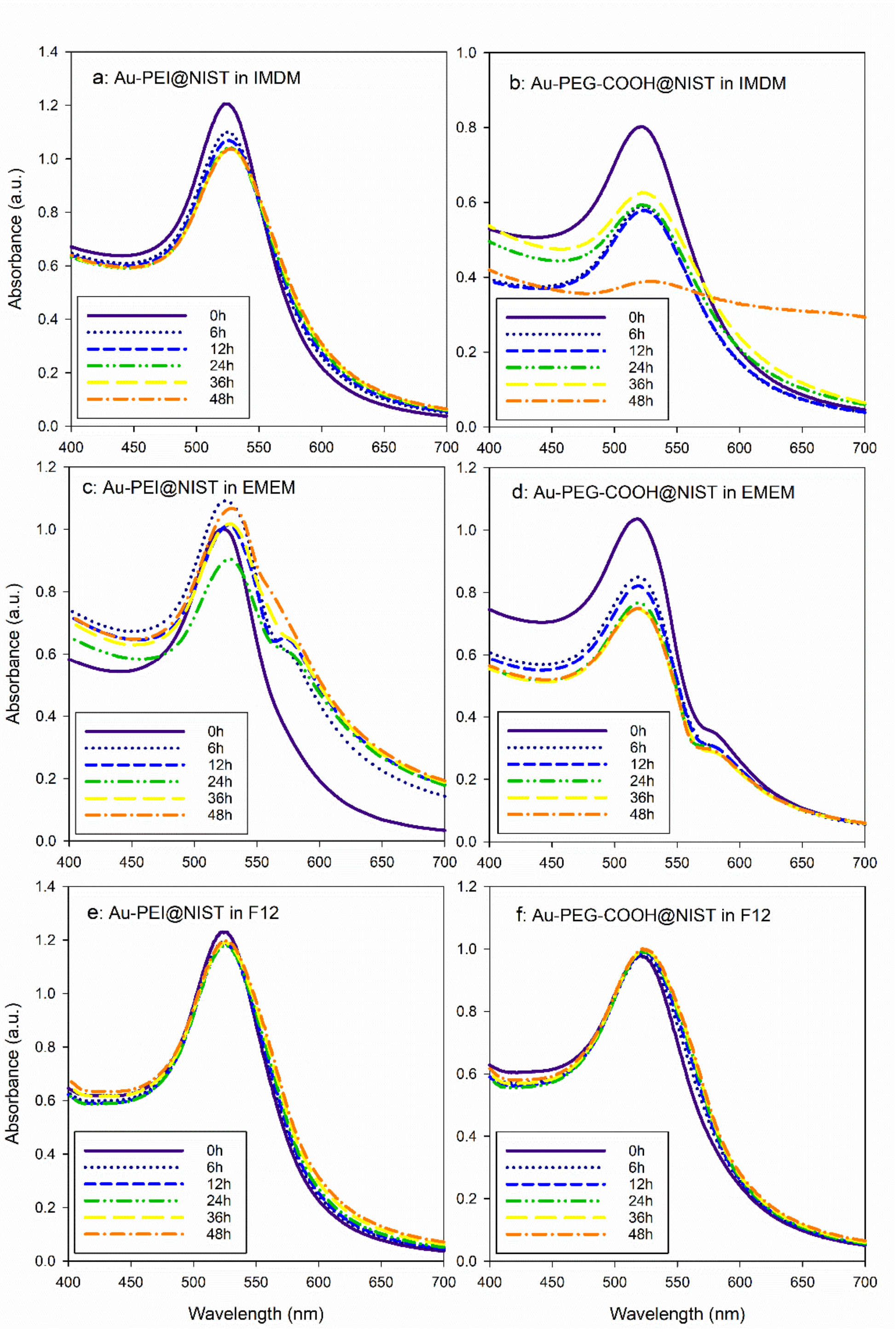

Figure S8. Colloidal behavior of Au-PEI@NIST and Au-PEG-COOH@NIST in (a, b) IMDM, (c, d) in EMEM, and (e, f) in F12.

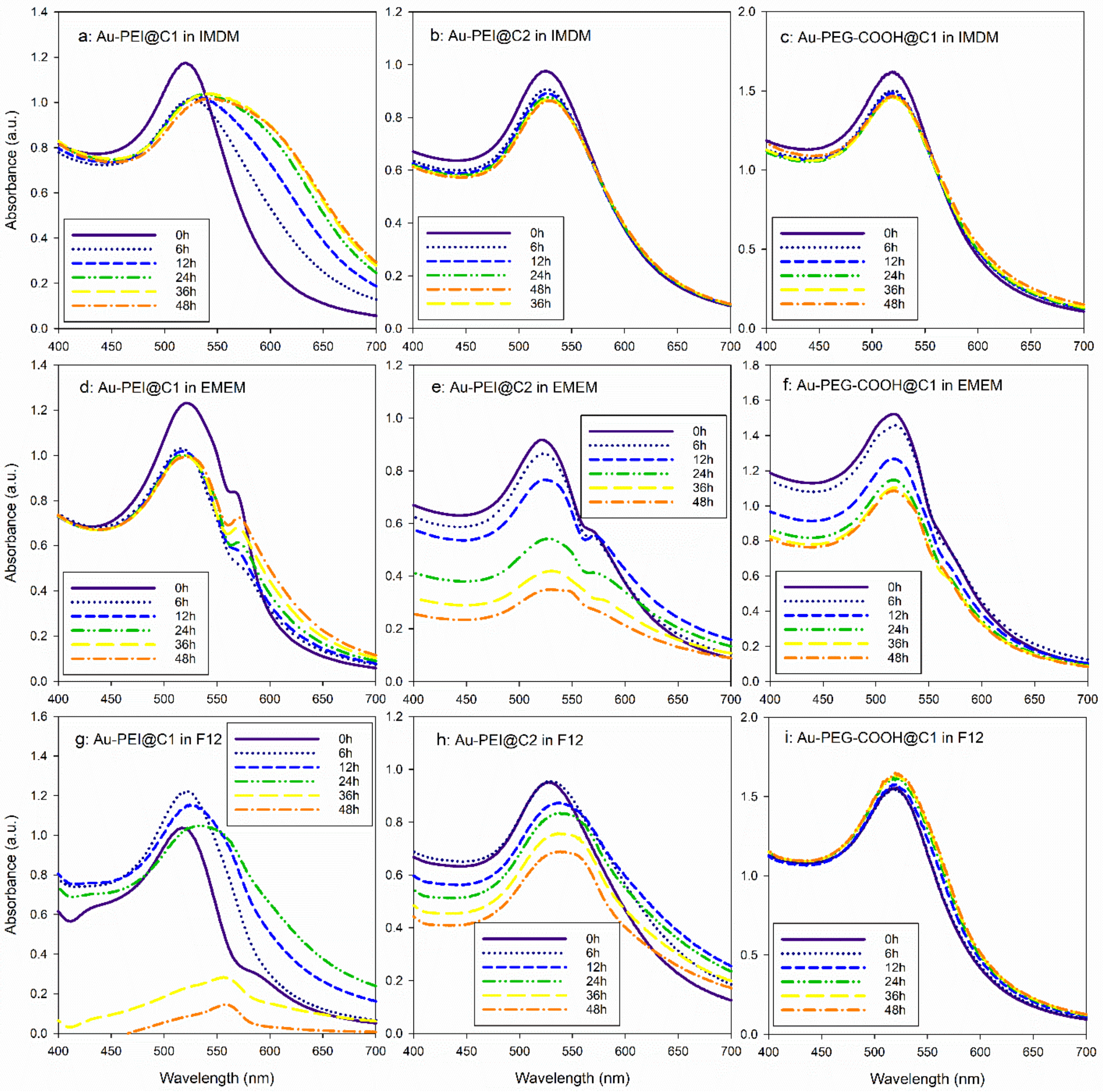

Figure S9. Colloidal behavior of commercially sourced AuNPs in IMDM (a, b, c), EMEM (d, e, f), and in F12 (g, h, i).

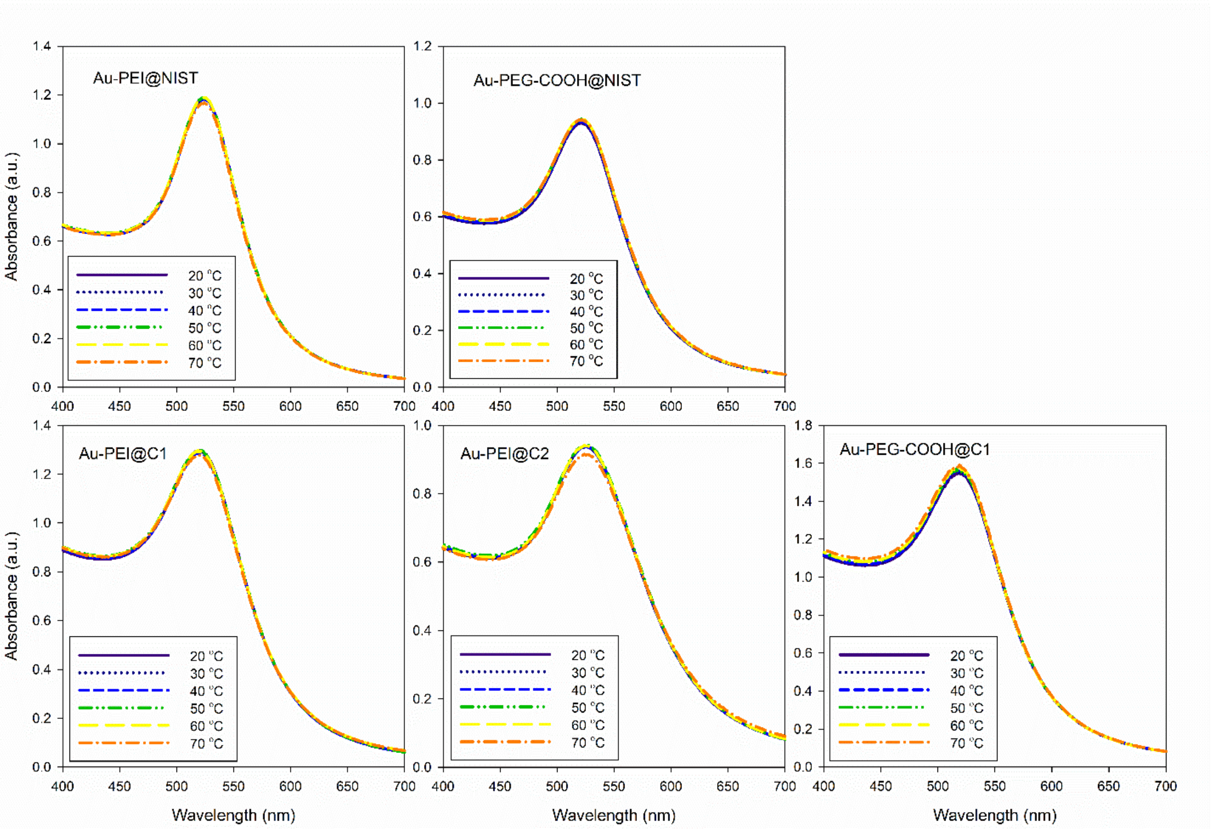

Figure S10. Thermal stability of all tested AuNPs over the temperature range from 20°C to 70°C.

Toxicity test of commercially sourced gold nanoparticles

Toxicity of commercially sourced gold nanoparticles was tested by incubating them with Melanoma (SK-MEL-28) cells for 24h. Cellular viability is shown in Fig. S11 overlayed with Au-PEI@NIST data. IC_{50} $_{Au\text{-}PEI@NIST}$=1.49±0.32 µg/mL ; IC_{50} $_{Au\text{-}PEI@C1}$=2.48±0.59 µg/mL ; IC_{50} $_{Au\text{-}PEI@c2}$=1.53±0.28 µg/mL

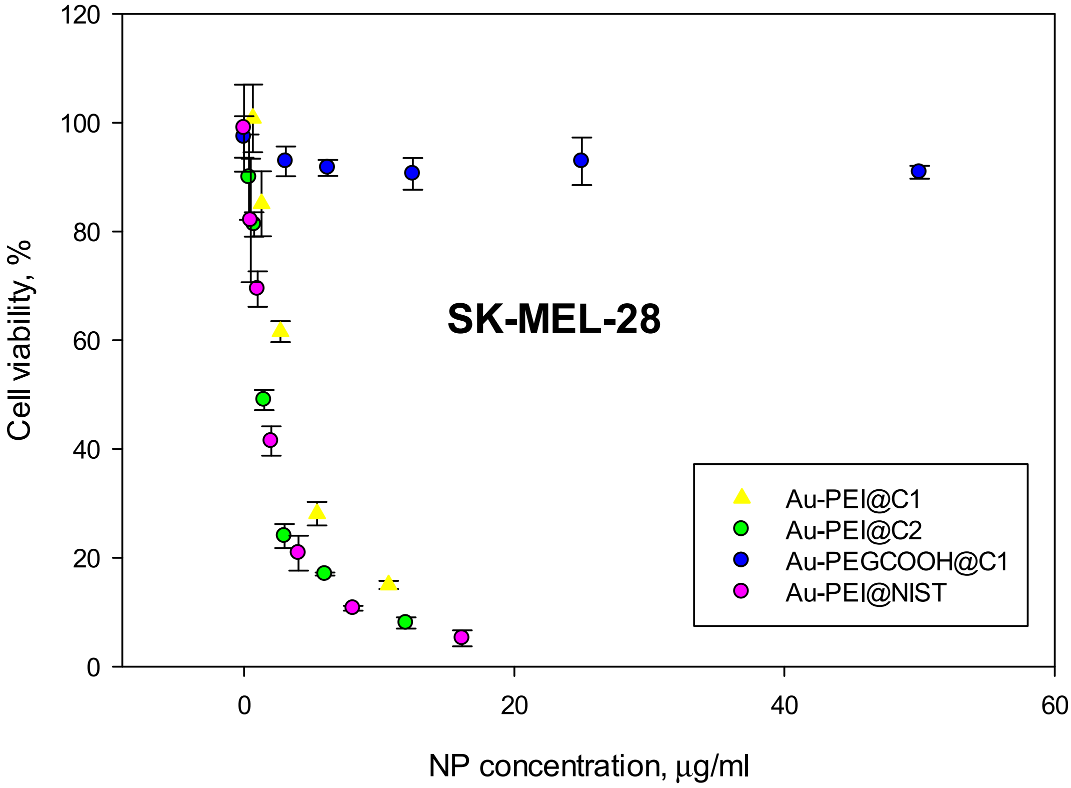

Figure S11. Cellular viability of skin cancer cells SK-MEL-28 following 24h exposure to Au-PEI and Au-PEG-COOH nanoparticles from two commercial sources C1 and C2 overlayed with Au-PEI@NIST data.

Toxicity of filtrate following centrifugal filtration

AuPEI@NIST nanoparticles were subjected to several cycles of centrifugal filtration as described in sec. 2.5. Toxicity of the filtrate was tested using CHO K1 cells by incubating them with serially diluted filtrates for 24h (Figure S12).

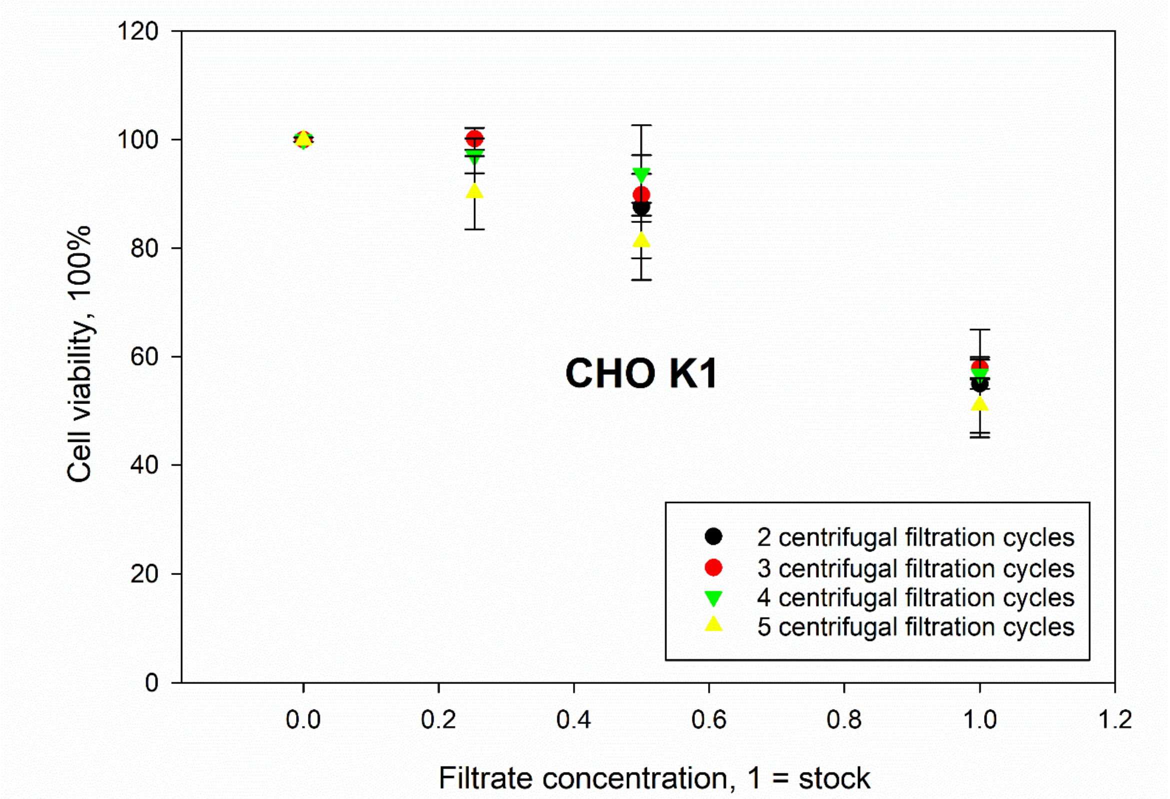

Figure S12. Toxicity of AuPEI@NIST filtrate tested with CHO K1 cells at three concentration levels – stock (1.0), 50% and 25% dilution with full growth media (0.5 and 0.25).
